# Genomic and immune profiling of breast cancer brain metastases

**DOI:** 10.1186/s40478-025-02001-3

**Published:** 2025-05-12

**Authors:** Amanda E. D. Van Swearingen, Marissa R. Lee, Layne W. Rogers, Alexander B. Sibley, Pixu Shi, Xiaodi Qin, Michael Goodin, Katelyn Seale, Kouros Owzar, Carey K. Anders

**Affiliations:** 1 Duke Center for Brain and Spine Metastasis, Duke Cancer Institute, Duke University, Durham, NC USA; 2 Duke Cancer Institute, Duke University School of Medicine, Durham, NC USA; 3 Department of Biostatistics and Bioinformatics, Duke University School of Medicine, Durham, NC USA; 4 Duke Cancer Institute, Duke University Hospital, Durham, NC USA; 5 Department of Biostatistics and Bioinformatics, Duke Center for Brain and Spine Metastasis, Duke Cancer Institute, Duke University School of Medicine, Durham, NC USA; 6 Department of Medical Oncology, Duke Center for Brain and Spine Metastasis, Duke Cancer Institute, Duke University, 10 Searle Center Drive, Campus Box 3881, Durham, NC 27710 USA

**Keywords:** Breast cancer, Brain metastasis, FFPE, Frozen, Intrinsic subtypes, Total RNA sequencing, Whole-exome sequencing, Immune landscape

## Abstract

**Background:**

Brain metastases (BrM) arising from breast cancer (BC) are an increasing consequence of advanced disease, with up to half of patients with metastatic HER2 + or triple negative BC experiencing central nervous system (CNS) recurrence. The genomic alterations driving CNS recurrence, along with contributions of the immune microenvironment, particularly by intrinsic subtype, remain unclear.

**Methods:**

We characterized the genomic and immune landscape of BCBrM from a cohort of 42 patients by sequencing whole-exome DNA (WES) and total RNA libraries from frozen and FFPE BrM and FFPE extracranial tumors (ECT). Analyses included PAM50 intrinsic subtypes, somatic mutations, copy number variations (CNV), pathway alterations, immune cell type deconvolution, and associations with clinical outcomes

**Results:**

Intrinsic subtype calls were concordant for the majority of BrM-ECT pairs (60%). Across all BrM and ECT samples, the most common somatic gene mutation was *TP53* (64%, 30/47). For patients with matched FFPE BrM-FFPE ECT, alterations tended to be conserved across tissue type, although differential somatic mutations and CNV in specific genes were observed. Several genomic pathways were differentially expressed between patient-matched BrM-ECT; MYC targets, DNA damage repair, cholesterol homeostasis, and oxidative phosphorylation were higher in BrM, while immune-related pathways were lower in BrM. Deconvolution of immune populations between BrM-ECT demonstrated activated dendritic cell populations were higher in BrM compared to ECT. Increased expression of several oncogenic preselected pathways in BrM were associated with inferior survival, including DNA damage repair, inflammatory response, and oxidative phosphorylation

**Conclusions:**

Collectively, this study illustrates that while some genomic alterations are shared between BrM and ECT, there are also unique aspects of BrM including somatic mutations, CNV, pathway alterations, and immune landscape. A deeper understanding of differences inherent to BrM will contribute to the development of BrM-tailored therapeutic strategies. Additional analyses are warranted in larger cohorts, particularly with additional matched BrM-ECT.

**Supplementary Information:**

The online version contains supplementary material available at 10.1186/s40478-025-02001-3.

## Introduction

As patients are living longer with metastatic breast cancer (MBC), the incidence of brain metastases (BrM) is increasing over time [3], with a recent meta-analysis citing an incidence of 31% for patients with HER2-positive (HER2 +) MBC, 32% for patients with triple negative MBC, and 15% for patients with hormone receptor (HR)-positive (HR +), HER2-negative (HER2−) MBC [41]. In fact, up to half of patients with metastatic HER2 + or triple negative breast cancer (TNBC) experience a central nervous system (CNS) recurrence [47, 64]. Despite recent advances in targeted therapies with increased efficacy in CNS disease [12], the prognosis of symptomatic BrM is poor, with shorter overall survival among MBC patients compared to those without BrM [9, 36]. As a result, there is need to better understand the biology and immune landscape surrounding BrM in MBC to identify potential therapeutic targets.

Several studies have performed sequencing of primary and BrM tissue from melanoma [21], lung cancer [69], breast cancer [7, 62, 65, 66, 75], and other solid tumor malignancies [7]. These studies demonstrate that BrM differ in biologically important ways compared to primary tumors and extracranial metastatic tissue, including different mutations and/or copy number variations (CNV) in clinically targetable genes such as *HER2* [7, 62, 66], *BRAF* [75], *PI3K/Akt* [7], *CDK* [66], and *ATM* [75]. A comprehensive genomic analysis (including mutational and CNV analyses, neoantigen prediction, and transcriptomic analysis) based on whole-exome and RNA sequencing of TNBC BrM and primary tumors showed BrM harbored higher mutational burden and single nucleotide variants (SNV)-derived neoantigen expression with reduced immune gene signature expression relative to primary TNBC [65]. These findings support the exploration of immunomodulatory treatments, such as vaccine development, in TNBC BrM.

While advances in the field of BCBrM are being made, BrM remain incurable such that continued exploration of the immunobiology of this disease state to uncover future therapies is warranted. Thus, in this study, we provide a comprehensive description of the genomic and immune landscape of BrM across all BC inferred intrinsic subtypes, with both DNA and (total) RNA from frozen and FFPE BrM specimens, including some matched ECT and/or primary tumor to contrast with BrM. We also briefly compare results from frozen and FFPE tissue from the same specimens to differentiate processing methods from true biology and explore some comparisons among clinical subtypes. Our goal was to identify features of BCBrM that may be shared with matched ECT, as well as those that are unique to BrM themselves, within and across subtypes, to further dissect the immunobiology of BrM to reveal clinically relevant targets that may inform future treatment strategies.

## Materials and methods

### Patient cohort

The cohort includes 42 patients with MBC who underwent craniotomy at Duke University Hospital for standard of care BCBrM resection between 1988 and 2019 who also had available frozen and/or FFPE BCBrM tissue for sequencing (Table 1, Additional file 1: Supplementary Fig. S1). All intracranial tissues (patients (*n*) = 39, samples (m) = 40) and any patient-matched fresh-frozen blood DNA (patients (*n*) = 25, samples (m) = 26) were obtained from the Duke Brain Tumor Center Biorepository (BTBR, IRB Pro00007434) at Duke University. Patient-matched whole blood DNA samples were collected according to best practices to account for germline variation in tumor samples to improve somatic variant calling. ECT (*n* = 12, m = 13) were FFPE tissue blocks pulled from the surgical pathology archives at Duke or outside hospitals. Collection of tissue samples and clinical information was approved by the Duke University Institutional Review Board (Pro00104321). Patient clinical subtypes were annotated based on standard of care clinical biomarker testing results from brain lesions, if available; otherwise, the clinical subtype was based on testing from a patient’s ECT if available; otherwise, the subtype was based on the primary tumor.

**Table 1:** Patient cohort overview

Patient cohort	
	Patient # (% of *n* = 42) or Median (IQR)
Race	
Asian	1 (2.4%)
Black or African American	12 (28.6%)
Caucasian	29 (69.0%)
Ethnicity	
Non-hispanic	41 (97.6%)
Not reported/declined	1 (2.4%)
Sex	
Female	42 (100%)
Male	0 (0%)
Status	
Alive/unknown	5 (11.9%)
Deceased	37 (88.1%)
Median age	
At initial breast cancer diagnosis	48 (44.2, 54.5)
At BrM surgery	53 (47.2, 61.0)
Clinical subtype	
HR + HER2− (hormone receptor +)	13 (31.0%)
HER2 + (HER2 +)	18 (42.9%)
HR + HER2 +	8 (19.0%)
HR− HER2 +	10 (23.8%)
HR + HER2eq^1^	1 (2.4%)
HR− HER2− (triple negative)	7 (16.7%)
Unknown	3 (7.1%)
Clinical subtype source	
BrM	25 (59.5%)
Distant metastasis	7 (16.7%)
Breast/LN	2 (4.8%)
Unknown/NA	8 (19.0%)
Year of BrM surgery	Surgery # (% of *n* = 43)^2^
1988–1999	2 (4.7%)
2000–2009	10 (23.3%)
2010–2020	30 (72.1%)
Status of BrM at surgery	Sample # (% of *n* = 40)^3^
Newly diagnosed	32 (80.0%)
Recurrent	6 (15.0%)
Unknown	2 (5.0%)
Treatment status of BrM at surgery	
Treated	7 (17.5%)
Not treated	31 (77.5%)
Unknown	2 (5.0%)
Treatment before surgery of BrM	
Radiation therapy	
Yes	7 (17.5%)
WBRT	3 (7.5%)
SRS	3 (7.5%)
WBRT + SRS	1 (2.5%)
No	31 (77.5%)
Unknown	2 (5.0%)
Systemic therapy	
Yes	1 (2.5%)
No	37 (92.5%)
Unknown	2 (5.0%)
Prior surgery	
Yes	4 (10.0%)
Subtotal resection	1 (2.5%)
Gross total resection	1 (2.5%)
Biopsy	1 (2.5%)
Unspecified	1 (2.5%)
No	34 (85.0%)
Unknown	2 (5.0%)

Demographic, clinical subtype, and treatment history summary of the patient cohort

BrM: Brain Metastasis, ER: Estrogen Receptor, LN: Lymph Node, NA: Not available, PR: Progesterone Receptor, HER2: human epidermal growth factor receptor 2, HER2eq: HER2 equivocal, WBRT: Whole Brain Radiation Therapy, SRS: Stereotactic Radiosurgery, FFPE: Formalin-Fixed Paraffin-Embedded

^1^The one HR + HER2eq patient was included with the HR + HER2− patients for most analyses

^2^One patient had two asynchronous BrM resections roughly a year apart

^3^Specimens were collected from 43 surgeries across 42 patients. Of the 43 surgeries, three of the BrM specimens were not available for sequencing, resulting in 40 BrM specimens

### Tissue sample preparation

H&E-stained slides were prepared from patient-derived, frozen and/or FFPE BCBrM tissues. Slides were reviewed by a pathologist, and blocks containing ≥ 50% viable tumor were chosen for sectioning for sequencing. Loose sections were collected from selected BCBrM and all available ECT blocks and used for nucleic acid extraction.

### Total RNA sequencing

Extraction of RNA from frozen BCBrM was performed using the RNeasy® Plus Mini Kit (QIAGEN, CAT # 74,134, Germantown, MD) and extraction of RNA from FFPE BCBrM and ECT tissues was performed using the RNeasy® FFPE Kit (QIAGEN, CAT # 73,504, Germantown, MD), both according to the manufacturer’s protocol. Extracted total RNA quality and concentration were assessed on a 2100 Bioanalyzer (Agilent Technologies) and Qubit 2.0 (ThermoFisher Scientific). RNA sequencing (RNAseq) libraries were prepared using Illumina Stranded Total RNA Prep with Ribo-Zero Plus kit (Illumina, CAT # 20,040,529) and indexed using Illumina RNA UD Indexes Set A (Illumina, CAT # 20,040,553). All libraries were then pooled in equimolar ratio and sequenced on four lanes of an Illumina NovaSeq 6000 S4 flow cell, generating 150 bp PE reads. Sequence data was demultiplexed and fastq files were generated using Illumina Bcl2Fastq conversion software.

### Whole-exome sequencing

Extraction of DNA from the isolated tissue was performed using the Gentra® Puregene® Tissue Kit (QIAGEN, CAT # 158,066, Germantown, MD) and extraction of DNA from blood was performed using the Gentra® Puregene® Blood Kit (QIAGEN, CAT # 158,026, Germantown, MD), both according to the manufacturer’s protocols. Extracted DNA was quantified using Qubit 2.0 (Thermo Fisher Scientific). DNA-seq libraries were prepared for each sample using the Kapa Hyper Prep kit (Roche, Cat # KK8504) and indexed using IDT for Illumina DNA unique dual indexes (UDI) Set A. Final libraries were quality checked using 2100 Bioanalyzer (Agilent Technologies) and Qubit 2.0 (ThermoFisher Scientific) and pooled into batches of 12 (pre-capture pooling). Each pool of libraries was then hybridized with IDT xGen Exome Hybridization Panel V2 probes (IDT, CAT # 10,005,152) to capture and pull down the portion of the DNA-seq library representing the human exome. Final captured libraries were amplified, pooled into one library, and sequenced using six lanes of an Illumina NovaSeq 6000 S4 flow cell (batch one had two lanes and batch two had four lanes), generating 150 bp paired end reads. Sequence data was demultiplexed and fastq files were generated using Illumina Bcl2Fastq conversion software.

### Bioinformatics and biostatistics considerations

#### Analysis of RNA sequencing data

RNAseq data were analyzed through the following steps. The quality of the raw sequencing reads was evaluated and reported using FastQC v0.11.9 [1] and MultiQC v1.10.1 [18]. Sequences with adapter contamination and low-quality sequences were cleaned using Trimmomatic v0.39 [6]. The quality of the remaining sequences was reevaluated to guarantee minimum adapter contamination.

The raw sequencing reads were aligned to the reference genome using the STAR aligner v2.7.8a [17]. The aligned reads were then mapped to annotated genomic features, including genes and exons, using STAR’s built-in module. The human reference sequence (GRCh38.p13) and annotation GTF file (gencode.v38.primary_assembly.annotation.gtf) were obtained from GENCODE [29, 30]. Mapping quality was evaluated before any downstream analyses. The read level mapping quality was evaluated through STAR output, including the fraction of reads mapped to gene regions, ambiguous regions, non-feature regions or multiple loci. Likewise, the base level mapping quality was accessed through CollectRnaSeqMetrics from Picard Toolkits v2.23.8 [8].

#### Molecular inferred intrinsic subtypes (PAM50)

A molecular intrinsic subtype was inferred for each RNAseq sample using expression data from the PAM50 50-gene panel [60]. The subtyping was performed using the R Bioconductor package genefu v2.30.0 [24], selecting the “pam50.robust” parameter set, and opting to rescale expression values by the quantile (q = 0.05), as recommended by the developer. In addition, we performed Claudin-Low subtyping as implemented in the package genefu with the function “ClaudinLow” and the built-in training dataset [61]. Because we anticipated differences in expression by sample type, samples were subtyped separately: frozen BrM (*n* = 30, m = 31), FFPE BrM (*n* = 33, m = 34), and FFPE ECT (*n* = 12, m = 13). To confirm that subtype calls were not highly dependent on the identity or number of samples included, we compared the calls to those from subtyping runs with all tumor samples (m = 78) and grouped by sample storage (frozen BrM m = 31 and FFPE m = 47). Sankey alluvial plots were used to visualize variation in subtype calls for a given patient across sample types and implemented in R with the package ggsankey v0.0.99999 (https://github.com/davidsjoberg/ggsankey). To represent a patient’s PAM50 inferred subtype, calls from frozen BrM RNA samples were prioritized if available (*n* = 30), followed by calls from FFPE BrM (*n = *7) or FFPE ECT samples (*n = *2), in that order.

#### Analysis of whole-exome sequencing data

Whole-exome sequencing (WES) data were processed and analyzed with the following steps. First, raw sequences were mapped to the hg38 reference genome using the BWA-MEM v0.7.17 algorithm [44]. The reference genome was obtained from the publicly available GATK resource bundle v0 (Homo_sapiens_assembly38.fasta; https://gatk.broadinstitute.org/hc/en-us/articles/360035890811-Resource-bundle). Then, aligned BAM files were preprocessed using Picard v2.23.8 and GATK v4.2.2.0 [16, 52, 74] to remove duplicate reads and perform base recalibration.

Somatic variant calling was performed by first constructing a panel of normals (PON), in the variant call format (VCF), of common artifactual and germline variant sites using all patient-matched blood samples (GATK v4.2.2.0 Mutect2, GenomicsDBImport, CreateSomaticPanelOfNormals). Somatic variants were then called in all tumor samples using GATK v4.4.0.0 Mutect2 with default parameters and an additional flag (–f1r2-tar-gz) for collecting F1R2 counts as input for LearnReadOrientationModel. Patient-matched blood samples (–input flag for normal BAM file and –normal-sample flag for specifying the normal library name) were used in combination with the PON VCF (–panel-of-normals flag) to call somatic variants in tumor samples. Initial callsets were prepared for filtering by first estimating the fraction of reads introduced by cross-sample contamination (GATK v4.4.0.0 GetPileupSummaries, CalculateContamination), and second, using the F1R2 counts collected by Mutect2 to calculate prior probabilities of single-stranded substitution errors prior to sequencing for each trinucleotide context (GATK v4.4.0.0 LearnReadOrientationModel). Finally, callsets were filtered (GATK v4.4.0.0 FilterMutectCalls) with default thresholds. Filtered callsets, including only SNVs and insertion/deletions (indels), were then annotated using Ensembl Variant Effect Predictor (VEP) v110.1 [53] and converted to the mutation annotation format (MAF) using vcf2maf v1.6.2.1 [37]. The maftools [51] R package was used to visualize and summarize somatic variant types across samples. Tumor mutational burden (TMB) was calculated as the total number of somatic mutations divided by the 34 Mb target exome capture size, following the maftools function tmb.

Oncoprint plots of 110 clinically relevant (CR) genes and the top 50 genes with the highest frequency of somatic alterations were generated for BrM and ECT samples. Differences in TMB between FFPE BrM and FFPE ECT patient-matched samples (*n = *6), and FFPE BrM and frozen BrM patient-matched samples (*n = *15) were tested using the Wilcoxon signed-rank test. Differences in TMB between PAM50 inferred subtypes within FFPE BrM (Basal: *n = *5, HER2-enriched: *n = *6, LumA: *n = *2, LumB: *n = *4), FFPE ECT (Basal: *n = *2, HER2-enriched: *n = *3, LumA: *n = *1), and frozen BrM (Basal: *n = *7, HER2-enriched: *n = *5, LumA: *n = *5, LumB: *n = *2) were tested using the Kruskal–Wallis test within each sample type. The Normal PAM50 inferred subtype group was dropped from these comparisons due to low sample size (m = 2). Differences in TMB between clinical subtypes within FFPE BrM and frozen BrM were also evaluated using Kruskal–Wallis tests; analyses were performed using clinical subtype determined in a BrM.

CNVs were called in all tumor samples (m = 76) using cnvkit v0.9.10 [71]. First, sequence-accessible coordinates were identified using the publicly available GATK hg38 reference genome (Homo_sapiens_assembly38.fasta) to estimate on- and off-target bin sizes (cnvkit access) and read depths from input recalibrated BAM files for all WES samples (cnvkit autobin). Coverage was then calculated from input BAM read depths within the given on- and off-target regions (cnvkit coverage). A pooled normal reference was constructed using all patient-matched blood samples (output from cnvkit coverage; cnvkit reference) and copy number segments were inferred for each tumor sample using cnvkit segment, with –threshold set to 0.00001 to lower the significance threshold for accepting breakpoints during segmentation, and with the –drop-low-coverage flag to drop very low coverage bins before segmentation. Segmentation outputs were converted to SEG format (cnvkit export seg).

Finally, the following GISTIC2 [55] analyses were constructed using BrM samples. The first was run across all BrM samples to identify regions of the genome that were significantly amplified or deleted. The analysis was also run for sample subsets representing each PAM50 subtype and each clinical subtype determined based on a BrM. SEG files for each BrM sample were concatenated into a TSV file either within each PAM50 inferred intrinsic subtype, clinical subtype, or across all BrM samples to create a single input for each GISTIC2 run. GISTIC2 was run using the hg38 reference (hg38.UCSC.add_miR.160920.refgene.mat) with default parameters, except for -conf, which was increased to 0.9 to increase the confidence level used to calculate regions containing a driver, and -genegistic, which was set to 1 to use the gene GISTIC algorithm for calculating the significance of deletions at a gene level instead of a marker level. Plots were generated to illustrate aberrant regions based on two significance thresholds: q values less than 0.25 (gray peaks) and 0.1 (red and blue peaks).

#### Differential gene expression

Differential gene expression based on sample type was analyzed within the framework of a negative binomial model using R [63] and the extension package DESeq2 v1.38.2 [49]. The following contrast was analyzed using patient-matched samples: ECT versus BrM in FFPE samples, with ECT as the baseline level, and controlling for patient identity. Genes were pre-filtered to select genes with more than five reads in a minimum of 20% of the ECT and BrM samples**.** False-discovery-rate (FDR) -adjusted *p* values, i.e., q values, were reported; genes with q value < 0.05 were considered significant. Gene set enrichment analysis (GSEA) was conducted on the same set of contrasting groups, first using 50 Hallmark pathways [45], and then with a set of 79 pathways of interest that were selected a priori and included pathways relevant to oxidative phosphorylation, MAPK interacting serine/threonine Kinase (MNK), cyclin dependent kinase (CDK), DNA damage repair, and immune signaling (Additional file 2). GSEA was performed using the R package fgsea v1.24.0 [39] with the permissible geneset sizes set to 15–500 and 3–1000 for the Hallmark pathways and the pathways of interest, respectively.

#### Immune cell fractions

To investigate immune cell infiltration, we used the CIBERSORTxFractions module within the CIBERSORTx software [59] to infer the relative fraction of 22 immune cell types based on gene expression data and the LM22 [59] signature matrix. Cell fraction differences between sample types in patient-matched samples (FFPE BrM and FFPE ECT, FFPE BrM and frozen BrM) were tested using the Wilcoxon signed-rank test and, to account for multiple testing, the resulting *p* values were adjusted using the Benjamini–Hochberg method [4]. Differences in cell fractions based on PAM50 intrinsic subtype or based on clinical subtype within FFPE BrM and frozen BrM were evaluated using Kruskal–Wallis tests; only clinical subtypes determined based on a BrM were used. Changes in relative cell fractions within selected cell types between FFPE BrM and FFPE ECT sample types and PAM50 classification of the RNA sample were investigated as well. For instance, variation within dendritic cell populations was calculated as the fraction of each dendritic cell type (activated and resting) over the sum of all dendritic cells; the same procedure was used to investigate macrophages (M0, M1, and M2), T cells (CD4 memory activated, CD4 memory resting, CD4 naive, CD8, follicular helper, gamma delta), and dendritic cells (activated and resting). Changes in the ratio of M1:M2 macrophage fractions based on sample type and PAM50 classification of the RNA sample were also investigated. To account for zero values, M1:M2 ratios were transformed using the formula atan( log2(M1/M2))/pi*2. Changes in M1:M2 ratios between sample types in patient-matched samples (FFPE BrM and FFPE ECT, FFPE BrM and frozen BrM) were tested using the Wilcoxon signed-rank test. Within each sample type, differences in M1:M2 ratios based on PAM50 classification were tested using the Kruskal–Wallis test.

#### Time-to-event analyses

Cox proportional hazards (PH) regression was used to perform time-to-event analyses on the following outcomes: (i) initial diagnosis to craniotomy, (ii) craniotomy to death/last follow-up, and (iii) initial diagnosis to death/last follow-up. Time-to-event analyses were performed using the R extension package survival v3.4.0 [72]. The association of PAM50 inferred subtype with the times to these events was investigated using the patient’s inferred subtype as inferred from RNA expression and selected based on sample type priority (frozen BrM > FFPE BrM > FFPE ECT) and availability as described above. The associations between clinical subtypes with the times to these events were also investigated; analyses were performed using clinical subtype determined based on a BrM. A two-part analysis was used to examine associations between geneset enrichment in FFPE BrM and the time from craniotomy to death/last follow-up. First, Cox PH regression was used to estimate the association between variance-stabilized expression of each gene with survival, controlling for the patients’ inferred subtypes. Second, the Wald statistics for the gene effect from each of the Cox PH models were used to run GSEA, as described above, on nine Hallmark pathways, (Cholesterol Homeostasis, DNA Damage Repair, IL6 JAK STAT3 Signaling, IL2 STAT5 Signaling, Inflammatory Response, Alpha Response, Interferon Gamma Response, Oxidative Phosphorylation, Reactive Oxygen Species Pathway), preselected based on their involvement in immune signaling or because they were previously reported to be prognostic [23, 43, 77]. Quantile–quantile plots (qq-plots) were generated for the Cox PH models. Kaplan–Meier (KM) plots were generated to illustrate selected associations with time to event outcomes. For the purposes of illustrating associations with continuous variables, covariates were dichotomized at the median.

## Results

### Patient and sample cohort characteristics

Patient cohort demographics are listed in Table 1. There were 42 breast cancer patients with BrM included in this analysis, of which 39 patients contributed 40 BrM specimens sufficient for sequencing procedures. Of the 42 patients, approximately two-thirds were Caucasian (*n = *29, 69.0%), with Black/African American (*n = *12, 28.6%) and Asian (*n = *1, 2.4%) races also represented; 41 (97.6%) identified as non-Hispanic, with one (2.4%) declining to report ethnicity. All 42 patients were female, and the median age at initial breast cancer diagnosis and BrM surgery were 48 and 53 years old, respectively. All clinical subtypes, including ER/PR (HR) + HER2− (*n = *13, 31.0%), HER2 + (*n = *18, 42.9%), HR−HER2− (*n = *7, 16.7%) were represented, along with 1 HR + HER2-equivocal patient who was combined with the HR + HER2− patients for applicable analyses (total HR + HER2− in analyses *n = *14). Clinical subtype assignment of BrM was determined by clinical testing of the BrM if available, which was true for the majority of cases (*n = *25, 59.5%). In the absence of information from the BrM, clinical subtype was annotated based on ECT (*n = *7, 16.7%) or primary tumor (*n = *2, 4.8%); otherwise, it could not be determined (Unknown/NA *n = *8, 19.0%) (Table 1). Of the 40 BrM specimens sufficient for sequencing, most were newly diagnosed at the time of craniotomy (32 surgeries, 80.0%), with several recurrent (6 surgeries, 15.0%) and unknown (2 surgeries, 5.0%). BrM were largely untreated (31 surgeries, 77.5%), with the known treated BrM (7 surgeries, 17.5%) having received prior radiation therapy (3 WBRT, 3 SRS,1 WBRT and SRS). Four BrM specimens (10.0%) were from patients who had undergone a prior surgery (Table 1). Treatments received following craniotomy are listed in Additional file 1: Supplementary Table S1. Most patients in the cohort were deceased (*n = *37, 88.1%), with the remainder (*n = *5) lost to follow up (Table 1). Median overall survival time from diagnosis to death/last follow-up was 74.3 months (95% confidence interval: 55.6, 122.0).

Tissue specimens from the 42 patients were obtained and sequenced for this study, specifically frozen BrM, FFPE BrM, FFPE ECT. In addition, patient-matched blood DNA samples were collected to account for germline mutations in tumor samples and to improve inference of somatic alterations. Summaries of the specimen tissue types and sequencing types are in Additional file 1: Supplementary Table S1, with the patient specimen breakdown, subsequent sequencing, and resulting pairs/triplets further illustrated in Additional file 1: Supplementary Fig. S1. Of the 42 patients, 30 patients yielded 31 frozen BrM specimens, 36 patients yielded 37 FFPE BrM specimens, 12 patients yielded 13 FFPE ECT specimens, and 25 patients yielded 26 patient-matched blood DNA specimens. One patient contributed BrM specimens from two craniotomies; however, only samples derived from the first craniotomy were included in patient-matched analyses. One patient contributed two ECT specimens, one from breast tissue and one from an unspecified location; however, only the breast specimen was included in patient-matched analyses. These excluded specimens are referred to hereafter as “secondary BrM” or “secondary ECT” specimens (Supplementary Fig. S1) and were excluded from analyses of TMB, DE, and immune cell populations. Patient-matched DNA blood samples used to call somatic variants were only available for a subset of patients (*n = *25). CNV analyses used all tumor DNA samples regardless of whether a patient-matched blood DNA sample was available.

### Intrinsic and clinical subtype classifications and concordance

**Fig. 1:**
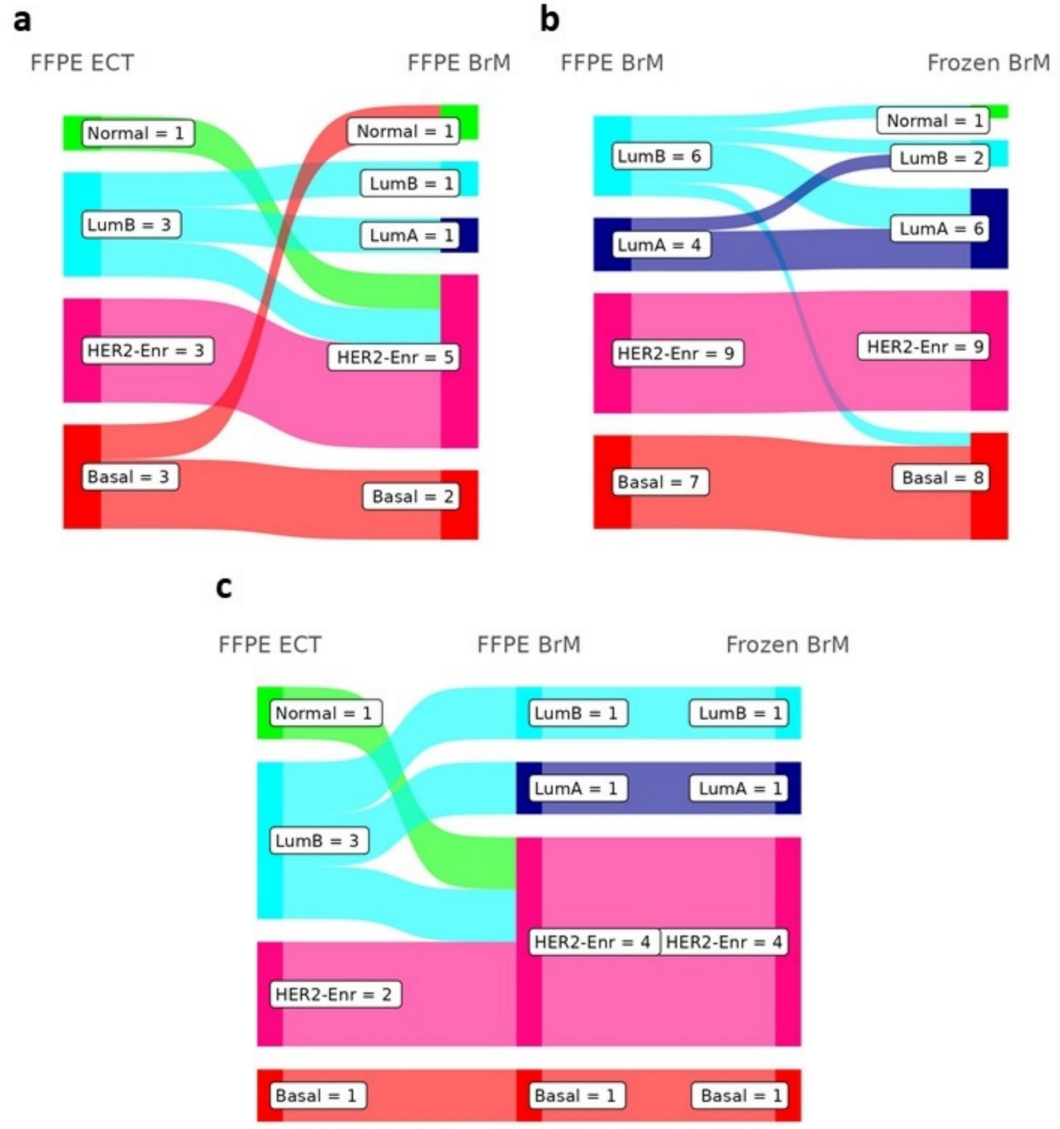
Intrinsic subtype concordance between extracranial tumors and brain metastases. Concordance of PAM50 inferred intrinsic subtypes in patients between matched **a** FFPE ECT and FFPE BrM (*n* = 10 patients), **b** FFPE BrM and Frozen BrM (*n* = 26), and **c** FFPE ECT, FFPE BrM, and Frozen BrM (*n* = 7). For the patient that contributed two ECT FFPE samples, only the ECT from breast tissue (PAM50 Normal) is considered in panels a and c; the other ECT sample was classified as HER2-Enr, matching the FFPE BrM from that patient. For the patient that contributed two FFPE BrM and two Frozen BrM, all samples were classified as Basal

We performed intrinsic molecular subtype analyses using RNAseq data based on the relative expressions of the 50-gene profile of samples (Additional file 1: Supplementary Fig. S2a) to determine the PAM50 inferred intrinsic molecular subtype for each sample type (Additional file 1: Supplementary Fig. S2b). We assessed subtype concordance within patients with multiple sample types. Matched FFPE ECT and FFPE BrM samples were largely concordant (*n = *6/10 patients, 60%, Fig. 1a). One of the four non-concordant patients contributed a second ECT from an unspecified location (not shown in Fig. 1); in sum, the patient contributed a HER2-Enriched (HER2-Enr) FFPE BrM, a Normal-like (Normal) FFPE ECT from breast tissue, and a HER2-Enr FFPE ECT from an unspecified location. The 3 patient-matched ECT samples classified as HER2-Enr had matched HER2-Enr FFPE BrM, and two of the three Basal-like (Basal) ECT were concordant with their Basal BrM. Discordant pairs involved the Luminal B (LumB) or Normal subtypes: of the three ECT with LumB classification, only one had a corresponding LumB FFPE BrM, and the Normal ECT was matched to a HER2-Enr BrM. Patients with FFPE BrM and frozen BrM matched pairs (*n = *26 patients, Fig. 1b) were also largely concordant (*n = *20/26 patients, 76.9%). Discordant pairs were again among the pairs with a LumB subtype: only one of the six FFPE BrM classified as the LumB subtype were also called as LumB in the matched frozen BrM, with the remainder classified as Normal (*n = *1), Luminal A (LumA) (*n = *3), or Basal (*n = *1) in the frozen BrM. The remaining discordant pair was a LumA FFPE BrM with a LumB frozen BrM. In the 7 patients with triplet samples (8 FFPE ECT, 7 FFPE BrM and 7 frozen BrM samples, Fig. 1c), most cases were concordant (*n = *4/7 patients, 57.1%) across triplets, with 100% concordance between corresponding FFPE BrM and frozen BrM samples in those triplets. Observed discordance again involved the LumB and Normal subtypes in ECT samples.

We also investigated concordance within BrM specimens between the PAM50 inferred subtype and the BrM-determined clinical subtype (Additional file 1: Supplementary Fig. S3). A total of *n = *22 BrM had both an assigned PAM50 subtype and a known clinical subtype. There was considerable diversity between PAM50 and clinical subtype alignments, though as expected, all BrM called HER2-Enr by PAM50 were HER2 + clinically, if clinically known (*n = *7/7, 100%). Most Basal-like BrM were HER2− clinically, if known (3/5, 60%). Both LumB BrM with known clinical subtypes were HR + as were most (*n = *6/7, 85.7%) of the LumA BrM.

### Mutational and somatic copy number alterations through WES

**Fig. 2:**
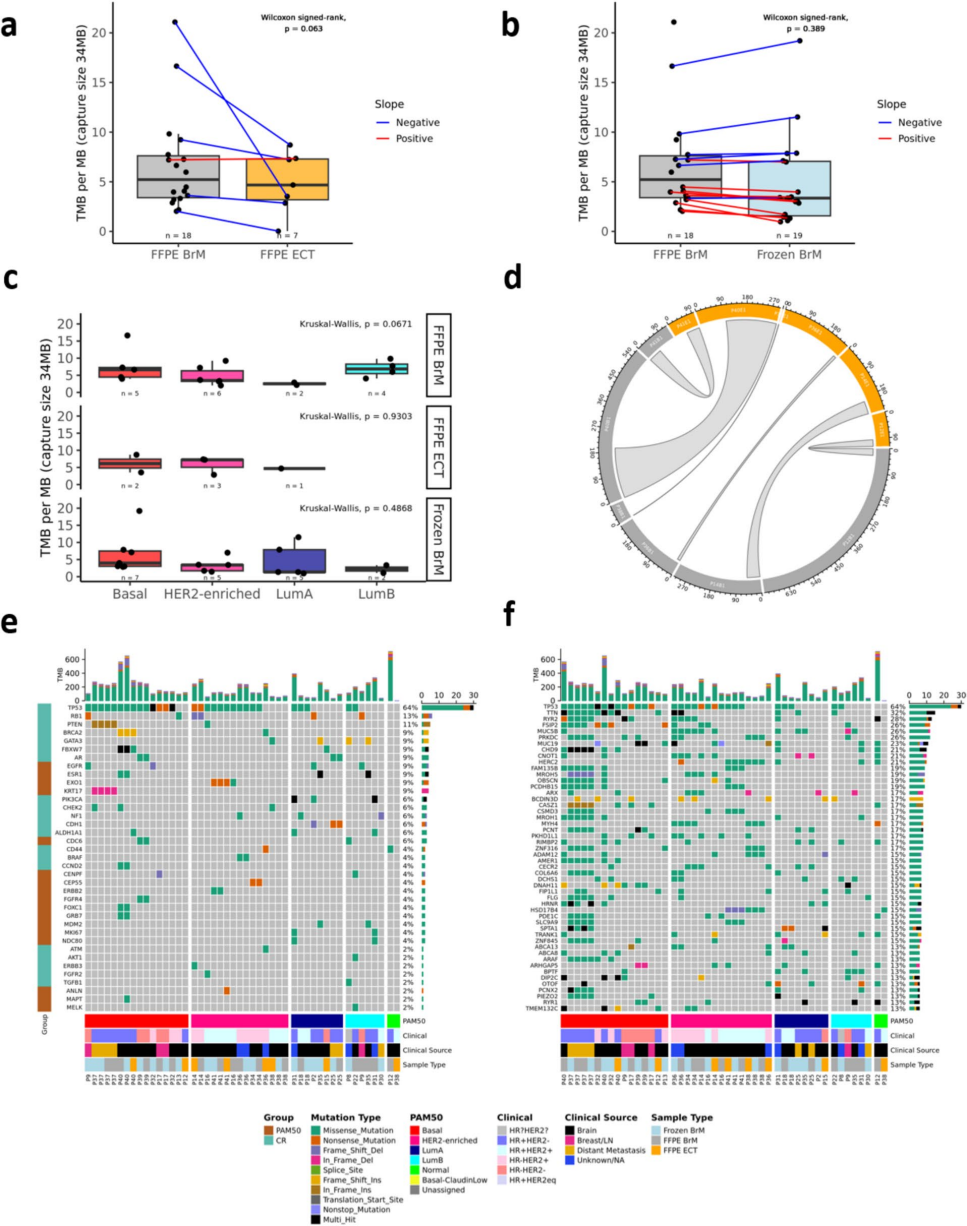
Mutational landscape of extracranial tumors and brain metastases. TMB for matched BrM-ECT and FFPE-frozen tumors with somatic variant calls, **a** paired FFPE BrM and FFPE ECT (*n* = 6 patients) and **b** paired FFPE BrM and frozen BrM (*n* = 15). Note that median lines shown in a and b are for all samples plotted. **c** TMB by PAM50 inferred intrinsic subtype for each sample type (FFPE BrM, *n* = 17; FFPE ECT, *n* = 6; Frozen BrM, *n* = 19; the two PAM50 Normal samples are not shown nor analyzed). **d** Circos plot demonstrating the total number of variants in each sample (by the width of the sample’s band in the outer ring) and the number of shared variants among the samples in patients with matched FFPE ECT and FFPE BrM (*n* = 6; by the width of the central bands). Secondary BrM and ECT samples are not shown nor analyzed in a-d. Oncoplots of **e** clinically relevant and **f** top 50 altered genes identified in tumor samples with somatic variant calls, including FFPE BrM (*n* = 18 patients, m = 19 samples), frozen BrM (*n* = 19, m = 20), and FFPE ECT (*n* = 7, m = 8). Top barcharts above e and f show tumor mutational burden (TMB) as the number of non-silent mutations by mutation type called across the genome. Clinical source refers to the location of the lesion assessed for clinical biomarkers (ER, PR and HER2) in that patient’s medical records used to assign the clinical subtype of the BrM

We compared TMB, estimated from the WES data, between two sets of patient-matched sample types. The first examined differences in TMB between patient-matched FFPE BrM and FFPE ECT (*n = *6). While FFPE BrM demonstrated a higher mutational load than FFPE ECT, there was no significant difference detected between tumor location and TMB (median mutations/MB: 8.22 in FFPE BrM vs. 5.38 in FFPE ECT, *p* = 0.063; Fig. 2a). We also examined differences in TMB between patient-matched FFPE BrM and frozen BrM. There was no significant difference detected between sample storage method and TMB (median mutations/MB: 4.06 in FFPE BrM vs. 3.47 in frozen BrM, *p* = 0.389; Fig. 2b). Differences in TMB were also examined across inferred intrinsic subtypes (Fig. 2c) within each sample type. No significant differences were detected within any sample type group (FFPE BrM, *p* = 0.0671; FFPE ECT, *p* = 0.930; frozen BrM, *p* = 0.487). Differences in TMB were also examined across clinical subtypes within FFPE BrM and frozen BrM samples (Additional file 1: Supplementary Fig. S4). For FFPE BrM, TMB varied by clinical subtype when restricted to those BrM whose subtype was determined by BrM (*n = *11, *p* = 0.018), with HR + HER2− samples having higher average TMB compared to other subtypes, though only one case was HR−HER2−. No significant differences by clinical subtype were detected within frozen BrM samples. An analysis of shared mutations within FFPE ECT and FFPE BrM patient-matched pairs (*n = *6) revealed varying degrees of mutational conservation within patients between ECT and BrM (Fig. 2d). Investigation of CR genes (Fig. 2e) and the 50 most frequently altered genes (Fig. 2f) in all samples with patient-matched blood DNA demonstrated that *TP53* was the most frequently altered gene (64% of samples), and the only gene altered in over half of samples (Additional file 3). Other genes were altered in a third or less of samples and were limited to a few patients’ tumors. As expected, across all somatic alterations, missense mutations were the predominant mutation observed (Additional file 4). For matched FFPE BrM-frozen BrM samples, alterations tended to be found in both samples, though some genes did show different mutational status between FFPE and frozen samples (e.g. *FIP1L1* missense alterations noted in FFPE BrM but not matched frozen BrM of 3 patients). Some patients’ tumors did demonstrate differential alterations between ECT and BrM in CR genes: one (P14) lost an *ERBB3* alteration from the ECT to the BrM; another (P36) lost *ATM*, *CD44*, and *BRCA2* and gained *BRAF* and *NF1* alterations in the BrM compared to ECT; a third (P40) gained alterations in *ESR1*, *CCND2*, *FOXC1* and *GRB7* in BrM that were not present in the ECT; a 4th patient (P41) gained an *ERBB2* and lost an *ANLN* alteration in the BrM that were present in the ECT.

**Fig. 3:**
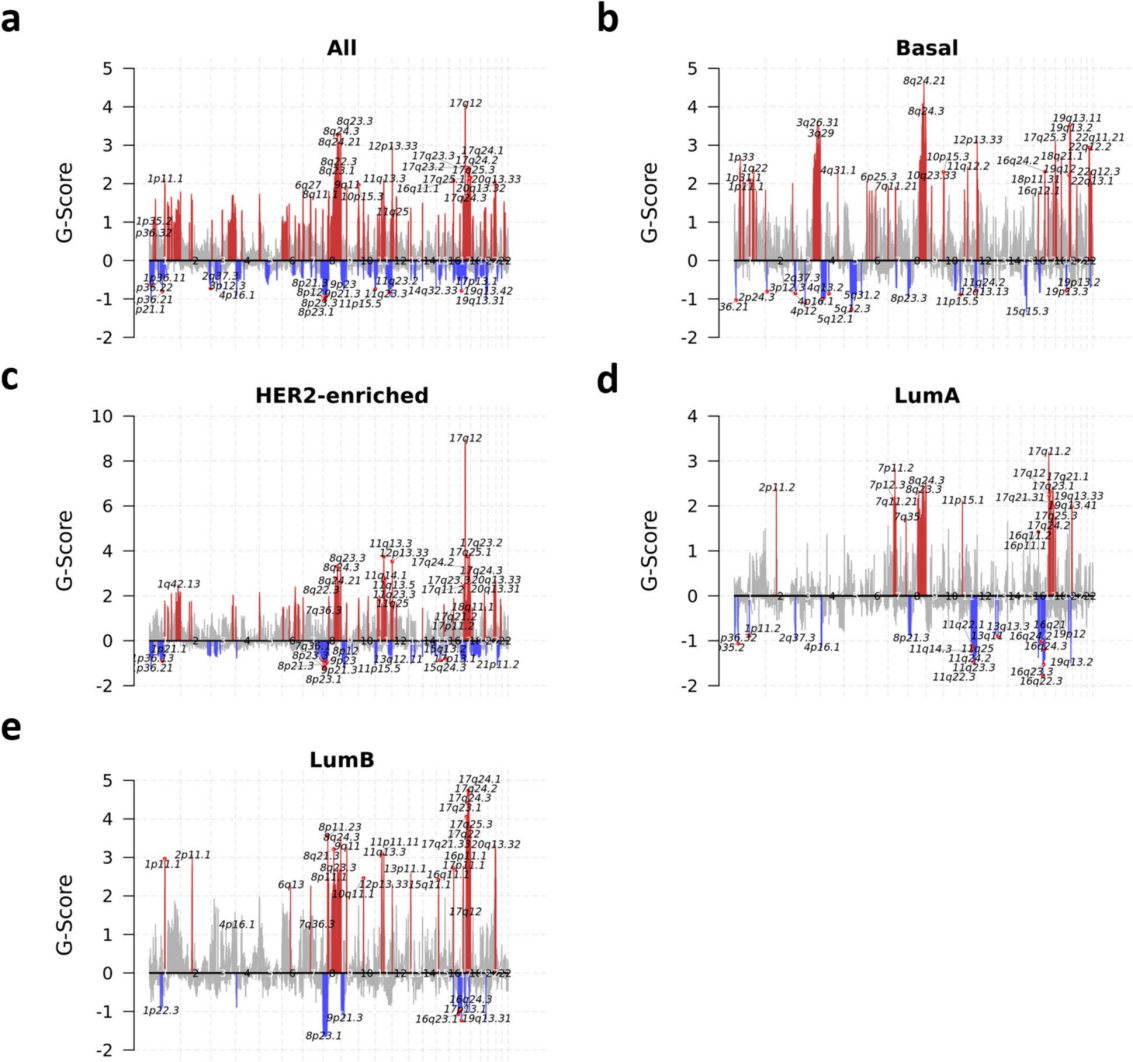

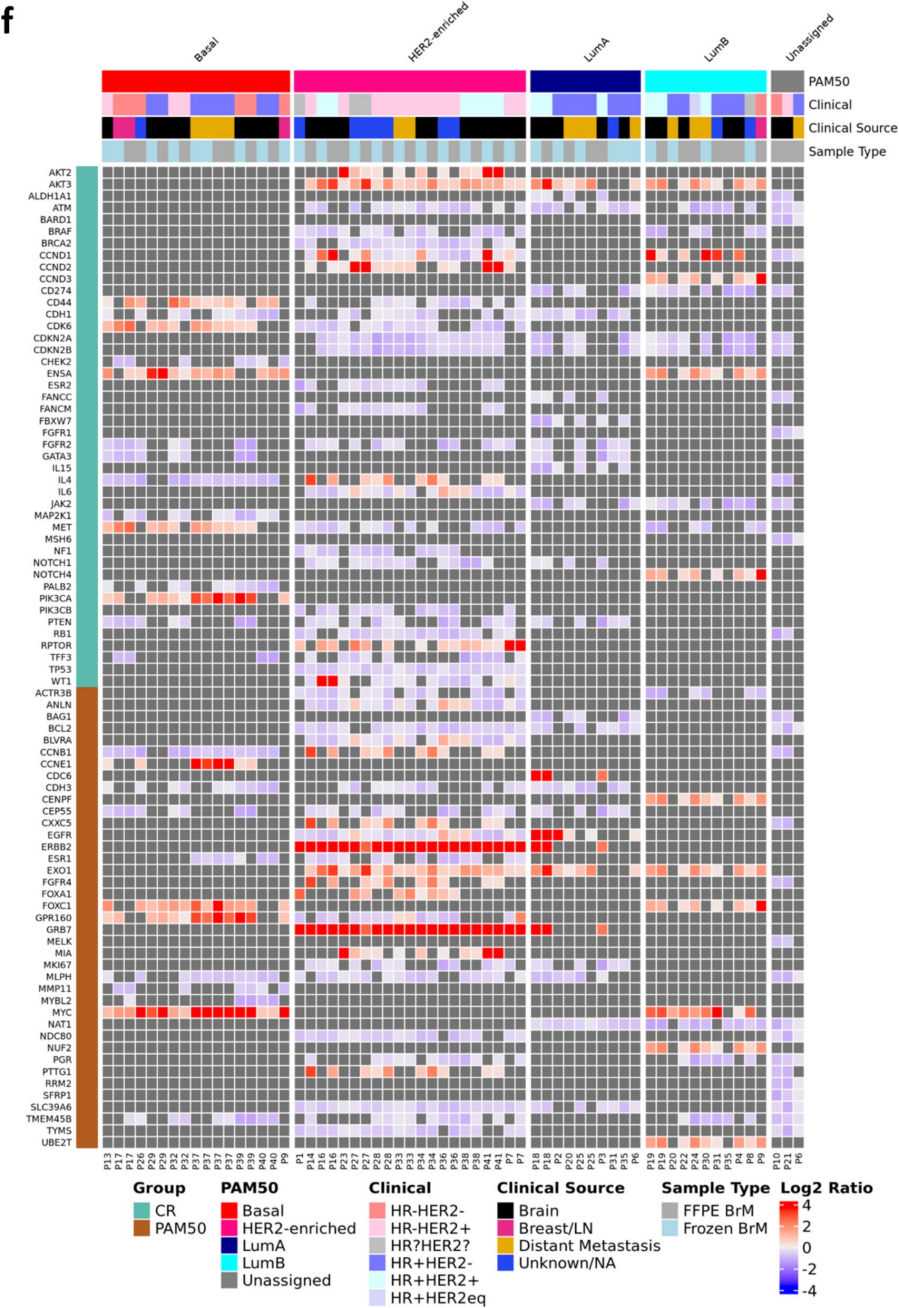
Copy number variants across BrM samples. Chromosomal map by GISTIC of CNV frequency and size by G-Score in **a** all BrM (*n* = 38 patients, m = 64 samples), **b** Basal (FFPE BrM *n* = 7, m = 8; frozen BrM *n* = 8, *m* = 9), **c** HER2-Enr (FFPE BrM *n* = 11, m = 11; frozen BrM *n* = 10, m = 10), **d** LumA (FFPE BrM *n* = 4, m = 4; frozen BrM *n* = 6, m = 6), and **e** LumB (FFPE BrM *n* = 7, m = 7; frozen BrM *n* = 4, m = 4). Gains/amplifications (red) are above the x axis, losses/deletions (blue) below the x axis, with significant (FDR < 0.10) variants colored and the chromosome arm region annotated; grey peaks indicate gains/amplifications with FDR < 0.25. Plots for PAM50 Normal and unassigned samples are not shown**, f** Heatmap of log2 copy number ratios in CR and PAM50 genes overlapping significantly amplified/deleted regions identified by GISTIC2 (FDR < 0.25) in FFPE BrM (*n* = 32 patients, m = 33 samples) and frozen BrM (*n* = 28, m = 29) by PAM50 inferred subtype. PAM50 Normals are not shown (FFPE BrM *n* = 1, m = 1; frozen BrM *n* = 1, m = 1) because no significantly amplified/deleted regions were identified by GISTIC2 among these samples. For cases where there was more than one GISTIC peak region associated with a given gene symbol and sample, the longest peak region was selected to be included in the heatmap. Clinical source refers to the location of the lesion assessed for clinical biomarkers (ER, PR and HER2) in that patient’s medical records used to assign the clinical subtype of the BrM

Analysis of copy number variations (CNVs) of BrM samples through GISTIC2 demonstrated several frequently gained and lost regions across the genome. Across all samples, 1p35-36, 8q23-24, 17q23-24, and 20q13 were frequently gained, and 1p36, 4p16, 8p, 9p, 11q, 17p, 19q were frequently lost (Fig. 3a). Within the Basal samples, BrM exhibited unique gains in 3q and 22q, and losses in 4p, 5q and 15q (Fig. 3b). HER2-Enriched samples featured unique amplifications in 1q, 20q, and 17q, including a large focal amplification at 17q12, where the *ERBB2* gene (HER2) is located, in addition to losses at 9p and 21p (Fig. 3c). LumA (Fig. 3d) and LumB (Fig. 3e) samples displayed fewer gained/lost regions overall, with LumA samples showing gains 7p and loss of 11q regions, and LumB samples exhibiting 8p gains and 9p21 and 16q losses.

Assessing gene-level copy number variations by CNVkit/GISTIC2 highlighted some expected and novel differences by PAM50 subtype in CR genes. As expected, the genes with high frequency of amplification across all subtypes include *MYC* (which correlates with the high frequency of regional amplifications at 8q24) in Basal and Luminal samples, and *CCND1* (on 11q13). Unsurprisingly, the genes *ERBB2* (HER) and *GRB7,* both of which are located on 17q, were amplified in every HER2-Enr sample (Fig. 3f). Relatively unique to Basal samples, *ENSA*, *CD44*, *GPR160*, *CDKN2A/B, FOXC1* and *PIK3CA* were frequently amplified, while *PTEN, GATA3, NOTCH1,* and *KRT14/17* were often lost. Somewhat restricted to HER2-Enr samples, beyond the expected *ERBB2* amplifications, were amplifications in *ANLN, BLVRA, and CCND2* in most samples and losses in *BRCA1/2, CCND1, CDK6* and *TP53*. *AKT3* was frequently gained in HER2-Enr, LumA and LumB samples, as was *CCND1* and *EXO1,* while *CDKN2A/B* were often lost. LumA samples demonstrated somewhat unique gains of *EGFR* and loss of *FANCC*, *JAK2*, *FGFR1*, and *SFRP1*, while LumB samples showed gains of *PALB2*, *CENPF*, *NUF2*, and *UBE2T* with losses of *ACTR3B* and *MKI67*.

Analyses of CNVs in BrM samples by clinical subtype were also performed (Additional file 1: Supplementary Fig. S5) using clinical subtypes as determined by BrM (*n = *22, Supplementary Fig. S5a). HR−HER2− BrM showed restricted gains in 6q15 and losses in 4q13, 11q24, and 13q14 (Supplementary Fig. S5b). HR−HER2 + specimens exhibited amplifications in several regions, including 17q12 (*ERBB2* locus) as well as gains at 8q23 and 12p13, with losses at 5q13 (Supplementary Fig. S5c). Amplifications in 2p25, 3q11, 5p15, 9q34, and 17q25 and losses in 3p14, 9p21, 11q23, 16q23, 17q12, 17q21, and 19q13 were observed in HR + HER2− BrM (Supplementary Fig. S5d), while the expected 17q12 amplification along with gains in 1q44, 8q23, 11q13, 12p13, 17q24, and 20q13 and losses in 1p36, 2q37, 7q35, 9p11, 11q24, 17p13, and 21p11 were observed in HR + HER2 + specimens (Supplementary Fig. S5e). Given the observed chromosomal variations, several clinically relevant genes exhibited amplifications and deletions, with some being somewhat restricted to specific subtypes, such as amplification of *ERBB2* and *GRB7* in HER2 + cases for both HR + and HR−, loss of *PTEN* in HR−HER2− samples, and loss of *BRAF* and *EGFR*, in HR + HER2 + cases with additional loss of *FGFR1* in both HR + HER2 + and HER2− cases.

### Gene and pathway expression by RNA sequencing

**Fig. 4:**
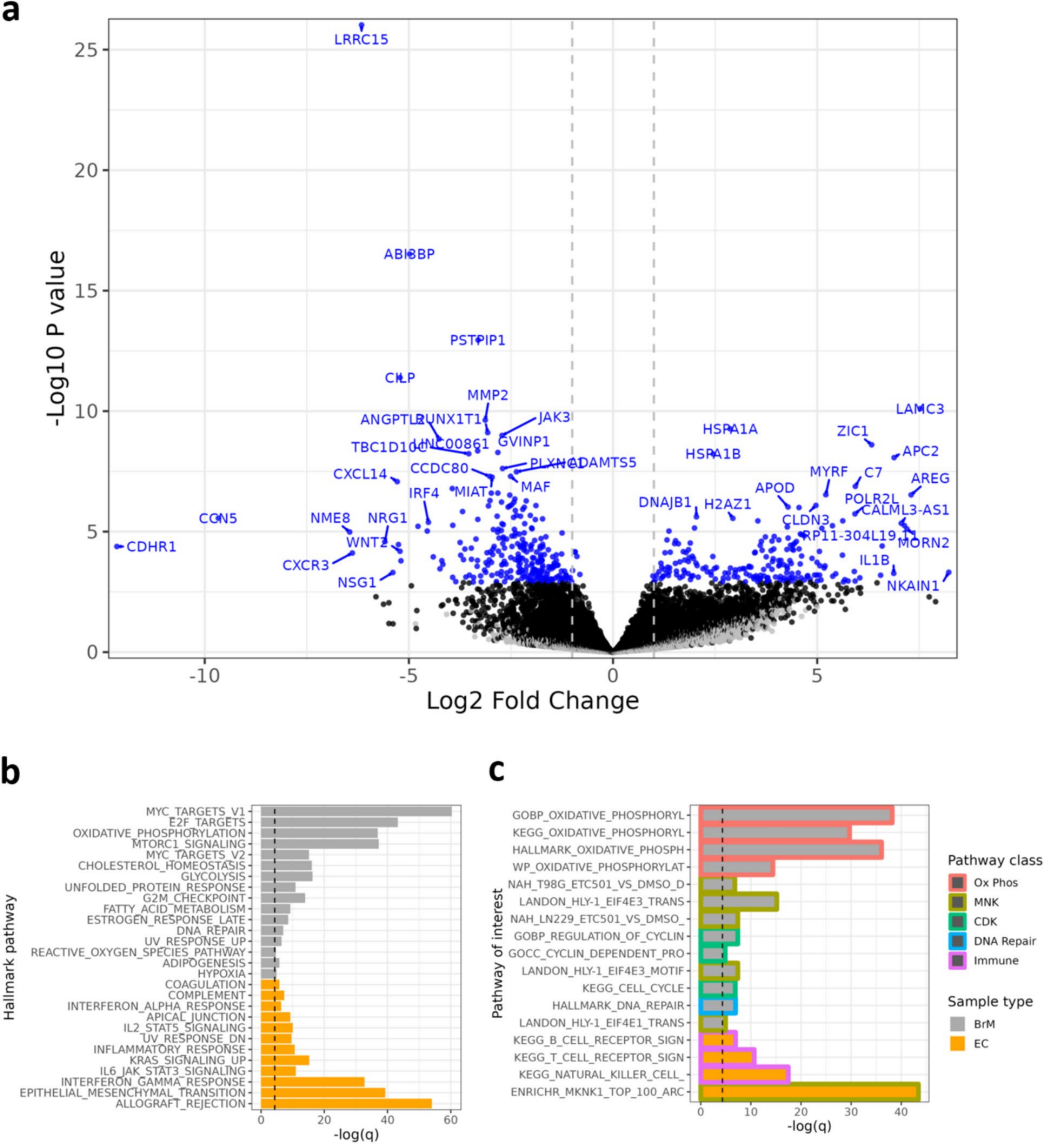
Differential gene and pathway expression in brain metastases compared to extracranial tumors. **a** Volcano plot of differentially expressed genes, **b** Hallmark pathways enriched in FFPE BrM compared to FFPE ECT, and **c** pathways of interest enriched in paired FFPE BrM-FFPE ECT samples (*n* = 8 patients). Excluded from analyses were a secondary ECT sample from a single patient and 2 patients with low quality samples and/or other outlier features. Genes were pre-filtered to those with more than five counts in a minimum of 20% of sample libraries per group

Differential gene expression was evaluated by utilizing total RNAseq data in matched FFPE ECT-FFPE BrM specimens. Patient-matched FFPE ECT and FFPE BrM samples were available from ten patients, however, two samples were excluded as outliers (Additional file 1: Supplementary Fig. S6) and the second FFPE ECT sample contributed by one patient was excluded. As a result, this analysis included eight matched FFPE ECT-FFPE BrM samples. In summary, 457 genes were differentially expressed between these 2 tissue locations, with 195 genes upregulated and 262 genes downregulated in the BrM tissues compared to ECT (Fig. 4a). Among the top significantly differentially expressed genes (DEGs), *LAMC3, APC2, IL1B, AREG, MORN2, HSPA1A/B, APOD, MYRF* and *DNAJB1* were higher in BrM compared to ECT, while *LRRC15, CDHR1, CON5, CSCR3, NSG1, BRG1, WIN2, CXCL14, ANGPTL2* were lower in BrM. The top 50 increased and top 50 decreased DEGs in the FFPE BrM samples compared to FFPE ECT samples are provided in Additional file 5. GSEA pertaining to the Hallmark pathways demonstrated increased expression of MYC target genes, gene targets of E2F transcription factors, and genes involved in oxidative phosphorylation in BrM compared to ECT. In parallel, pathways pertaining to allograft rejection, genes defining epithelial-mesenchymal transition, and genes up-regulated in response to interferon gamma and other inflammatory pathways were more highly expressed in ECT compared to BrM (Fig. 4b, Additional file 6). Looking at an a priori-selected set of pathways of interest from Hallmark pathways, KEGG, Gene Ontology (GO), and the literature [23, 42, 43], FFPE BrM tissues had increased expression of pathways related to oxidative phosphorylation, MAPK interacting protein kinase (MNK), cyclin dependent kinases (CDKs) and DNA damage repair, while FFPE ECT displayed higher expression of Immune pathway genes (Fig. 4c, Additional file 7).

### Immune landscape profiling via CIBERSORTx

**Fig. 5:**
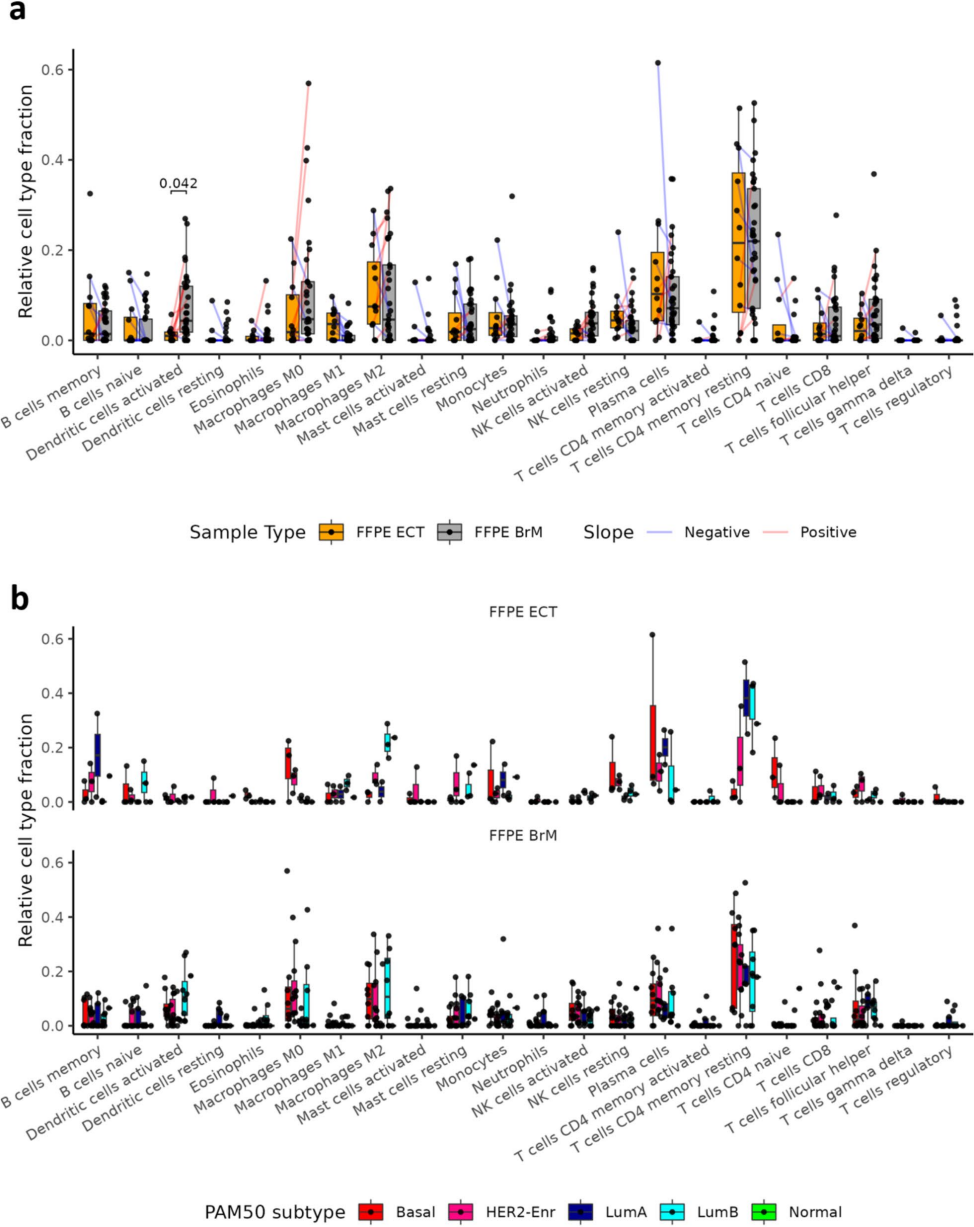
Deconvolved immune cell populations in extracranial tumors and brain metastases. **a** Fractions of immune cell populations inferred by CIBERSORTx in FFPE ECT (*n* = 12 patients, m = 12 samples) and FFPE BrM (*n* = 33, m = 33). Patient-matched FFPE BrM and FFPE ECT samples (*n* = 10) are connected by lines colored by slope direction. Significant differences (adjusted *p* < 0.05) are displayed between patient-matched FFPE BrM and FFPE ECT cell fractions based on Wilcoxon signed-rank tests with *p* values adjusted for testing multiple cell types. **b** Fractions of immune cell populations inferred by CIBERSORTx by intrinsic subtype within each tissue type (FFPE ECT *n* = 12, m = 12,; FFPE BrM *n* = 33, m = 33). No significant differences were detected between intrinsic subtypes within a given sample type using Kruskal–Wallis tests with *p* values adjusted for testing multiple cell types. Secondary BrM and ECT samples are not shown nor analyzed. PAM50 Normal sample (*n* = 1, m = 1) is shown in panel b, but not analyzed

The immune landscape of 22 immune cell types in BCBrM and matched ECT was investigated through CIBERSORTx deconvolution of RNAseq data. Comparing ten patient-matched FFPE ECT and FFPE BrM, only active dendritic cells showed differential fractions, being higher in the BrM compared to ECT (p_adj_ = 0.04, Fig. 5a). Comparing 26 patient-matched FFPE BrM and frozen BrM, M2 macrophages (p_adj_ = 0.01) and neutrophils (p_adj_ = 0.01) were higher in frozen BrM compared to FFPE BrM (Additional file 1: Supplementary Fig. S7a). After adjusting for multiple testing across cell types, no significant differences in cell fraction based on PAM50 intrinsic subtypes were detected within a given sample type (FFPE ECT, FFPE BrM, or frozen BrM) (Fig. 5b, Additional file 1: Supplementary Fig. S7b). Investigation of changes in relative cell fractions within selected cell types (i.e. dendritic cells, macrophages, and T cells), between FFPE BrM and FFPE ECT sample types and PAM50 classification of the RNA sample did not reveal strong differences by sample type within PAM50 status. However, inferences were limited by small sample sizes within each inferred subtype group (Additional file 1: Supplementary Fig. S8a–c). M1:M2 ratios were detected as lower in FFPE BrM as compared to patient-matched FFPE ECT based on 8 sample pairs with a non-zero M1 or M2 fraction (*p* = 0.007, Additional file 1: Supplementary Fig. S9a). No significant differences in M1:M2 ratios based on inferred intrinsic subtypes were detected within a given sample type (FFPE ECT, FFPE BrM, or frozen BrM, Additional file 1: Supplementary Fig. S9b–d).

Differences in inferred cell fractions by CIBERSORTx were also examined across clinical subtypes within FFPE BrM or Frozen BrM samples with clinical subtype determined by BrM testing (Additional file 1: Supplementary Fig. S10); however, no significant differences were detected after adjusting for multiple comparisons.

### Time-to-event analyses

**Fig. 6:**
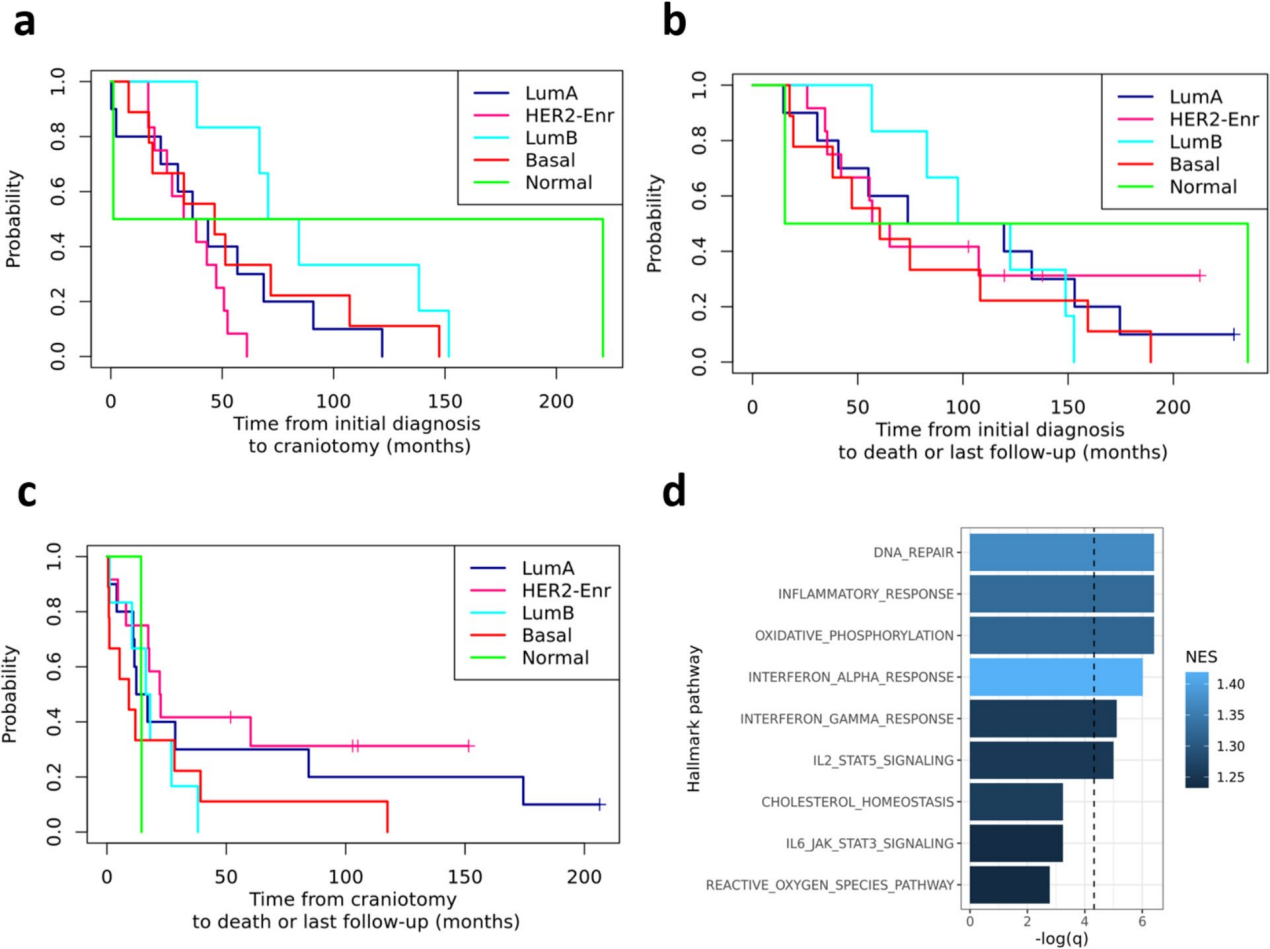
Clinical outcomes of patient cohort. Kaplan-Meyer plots of times to events (months) by inferred intrinsic subtype for time from **a** initial breast cancer diagnosis to craniotomy (*p* = 0.042, *n* = 39), **b** initial BC diagnosis to death or last follow-up (not significant) and **c** craniotomy to death/last follow up (not significant). **d** Association of expression of nine selected HALLMARK pathways of interest and survival from craniotomy to death/last follow-up in 33 FFPE BrM samples from 33 patients. PAM50 Normal BrM were excluded due to small sample size. For the patient with two craniotomies, data from the first craniotomy was used. Normalized enrichment score (NES) from geneset enrichment analysis. Pathways are ordered by adjusted P values. Positive values indicate that gene expression increases the hazard ratio (decreases survival time)

Utilizing clinical annotation of patient specimens, the outcomes of the patient cohort were assessed. For the patient with two asynchronous BrM that were resected and sequenced, the date of the first craniotomy was used for time-to-event calculations. The median length of time between a patient’s initial BC diagnosis to craniotomy of the sequenced BrM was 43.35 months and varied by inferred intrinsic subtype (*p* = 0.042; LumA (*n = *10) subtype 40.15 months, LumB (*n = *6) 77.50 months, HER2-Enr (*n = *12) 35.50 months, Basal (*n = *9) 46.60 months, and Normal (*n = *2) 111.0 months (Fig. 6a). Median time from initial diagnosis of BC to death/last follow-up was 74.30 months for the overall cohort, and did not vary by inferred subtype (*p* = 0.85, Fig. 6b). Median survival from craniotomy to death/last follow-up was 16.65 months, and similarly did not significantly differ by inferred subtype (*p* = 0.32, Fig. 6c). We explored whether expression of nine preselected Hallmark pathways were associated with time from craniotomy to death/last follow-up in patients with FFPE BrM (Fig. 6d). Six enriched pathways showed an association with worse survival outcome: DNA damage repair (Additional file 1: Supplementary Fig. S7a), inflammatory response (Additional file 1: Supplementary Fig. S7b), oxidative phosphorylation, interferon alpha response, interferon gamma response, IL2/STAT3 signaling, and cholesterol homeostasis. A caveat of this analysis is that we observed inflated *p* values from the Wald statistics of the gene-level time-to-event Cox PH models (Additional file 1: Supplementary Fig. S8).

We also tested for an association between clinical subtype as determined in BrM (*n = *25) and the times to these events (Additional file 1: Supplementary Fig. S13). No significant association was detected between clinical subtype and time from initial diagnosis to craniotomy (Additional file 1: Supplementary Fig. S13a). Time from initial diagnosis to death or last follow-up (Additional file 1: Supplementary Fig. S13b) and time from craniotomy to death or last follow-up (Additional file 1: Supplementary Fig. S13) varied by clinical subtype. HR−HER2− and HR−HER2 + cases had the shortest time from initial diagnosis to death/last follow-up, and HR−HER2− cases also had the shortest time from craniotomy to death/last follow-up compared to other clinical subtypes, while HR + HER2 + cases had the longest time for both intervals.

## Discussion

In this study, we have described the genomic and immune landscape of a cohort of 42 patients with available BCBrM tissues, along with some matched ECT, with a focus on recurrent alterations and potential biological differences between inferred intrinsic and clinical subtypes. We also report initial findings comparing frozen and FFPE processed BrM to identify potential technical artifacts that may influence clinically relevant interpretations of genomic data in the clinical setting. To our knowledge, this is the first study to analyze bulk total RNA sequencing and whole-exome DNA sequencing in a clinically-annotated set of both frozen and FFPE BrM, with some matched FFPE ECT, across all intrinsic and clinical subtypes of breast cancer with the goal of yielding a deeper understanding of the immunogenomics of BCBrM.

Comparison of patient-matched samples demonstrated that inferred intrinsic subtypes were largely concordant across intra-patient BrM and ECT tumor samples. While the majority of matched FFPE ECT and FFPE BrM exhibited the same intrinsic subtype, a significant portion (40%) of pairs were discordant, a rate similar to those found in prior studies within both intrinsic and clinical subtype comparisons [25, 32, 40, 57]. Of the discordant matched samples, half demonstrated HER2 enrichment in the BrM compared to the ECT, a phenomenon that has been previously reported in larger matched sample cohorts and has clinical implications [25, 32, 57, 62, 75]. Similar to some prior reports in primary BC tumors [38, 78], comparison of PAM50 intrinsic subtypes and clinical subtypes in BrM specimens demonstrated considerable heterogeneity between the two means of classifying BC subtypes, though major features like HER2 amplification/enrichment were consistent. Prior work has also shown that intrinsic subtypes can be more prognostic and predictive of therapeutic response and clinical outcomes in patients relative to immunohistochemical classifications alone [38, 60]. With the now well-demonstrated efficacy of HER2-targeting antibody–drug conjugates, such as trastuzumab deruxtecan (T-DXd) and tyrosine kinase inhibitors, notably tucatinib-based regimens, enrichment of HER2, and the ability of both immunohistochemical and molecular subtyping methods to accurately identify these cases in patients with BCBrM will alter the therapeutic approach and potential outcomes for these patients [10, 33, 58, 68].

Overall, BrM and ECT had similar TMB levels, though in matched samples, there was a trend towards higher TMB in BrM compared to ECT, a feature also found in prior larger studies in BrM compared to ECT/primary tumors [26, 31, 65], in BC metastases compared to primary BC tumors [15] and in metastatic compared to early BC primary tumors [2]. Within BrM, TMB did not significantly differ by intrinsic subtype, but did vary by clinical subtype in FFPE BrM, with HR + HER2− exhibiting higher average TMB than other subtypes, a feature which supports some prior reports of higher TMB in HR + HER2− and HR-HER2− metastatic BC compared to early stage or primary BC [5, 26], though this has not been found across every study [2]. Higher TMB may predict response to immunotherapies in a tumor agnostic fashion, as evidenced by the approval of pembrolizumab in this setting, thus opening another potential BrM-focused therapeutic avenue for patients [50, 67]. In this cohort and except for the tumor suppressor gene *TP53*, most gene-level somatic alterations were unique to individual patients’ BrM, as opposed to recurrent alterations across the cohort. However, while not recurrent between patients, many of the observed alterations were consistent with prior reports of acquired mutations in metastatic BC, those enriched in BCBrM, and those detected in ctDNA of CSF from patients with BCBrM, including *AKT1, BRAF, EGFR, ERBB2, ESR1, FGFR2, GATA3, PIK3CA, PTEN,* and *RB1* [5, 13, 14, 25, 26]. Albeit less frequent in our cohort, several additional gene alterations with known matched therapies were observed in individual patients, including *BRCA2* and *PTEN* alterations, opening up treatment opportunities with PARP inhibitors (i.e. talazoparib [48]) and AKT inhibitors (i.e. capivasertib [73]).

On the chromosome-level, the CNV landscape analysis of BrM yielded biologically-relevant and subtype-specific alterations including both gains and losses. Of note and across BCBrM subtypes, results illustrated gains in chromosomes associated with known driver genes, such as in 8q (*MYC*), 17q12 (*ERBB2, GRB7*), and 20q13 (*AURKA*) as well as losses in regions known to contain tumor suppressive genes (e.g. 9p21/*CDKN2A*, 13q14/*RB1,* 17p13/*TP53*, 17q21/*BRCA1*) that have previously been shown to be altered in metastatic BC and BCBrM [5, 26, 57]. Interestingly, prior work has illustrated that BC metastases are driven more by amplifications and deletions than by somatic mutations or rearrangements [26], thus further exploration of the impact of CNV on BrM is warranted [70].

When comparing BrM to matched ECT in our study, hundreds of individual genes were found to be differentially expressed, including some genes previously reported to be associated with BrM in BC, such as alphaB-crystallin (CRYAB) [76] and matrix metalloproteinase (MMP2) [54]. However, these differential genes did not contain the 4 genes (ARG2, SOX2, EGF, and NCAM1) previously identified in a larger cohort of matched BCBrM and primary BC as being up-regulated in BCBrM [27]. As it can be challenging to synthesize biology based on individual gene alterations, we elected to evaluate pathway alterations between BrM and ECT, yielding several interesting findings. Firstly, the oxidative phosphorylation pathway has previously been reported to be increased in melanoma BrM compared to non-CNS melanoma tumor tissue, and melanoma BrM that exhibit high oxidative phosphorylation signals also exhibit altered metabolism pathways dependent on glutamine [20]. Moreover, therapeutic targeting of the oxidative phosphorylation pathway with IACS-10759 or of glutamine metabolism with glutaminase inhibitor CB839 in mouse models of melanoma BrM improved survival and reduced BrM burden [20, 22]. This appears to be a BrM-specific phenomenon unrestricted from primary tumor type, opening the possibility of a pan-BrM therapeutic strategy [19–22, 79, 81]. In addition, CDK, mTOR, and DNA Repair pathways have also been previously shown to be enriched in BrM arising from BC and other solid tumors [7], and have become the focus of genomically-guided, prospective clinical trials in the BrM space (NCT03994796). Several clinical studies are also utilizing PARP inhibitors, either in combination with radiation and/or immunotherapy, to specifically treat patients with BCBrM (NCT04711824, NCT04837209, NCT05700721). Other groups have also illustrated the relative deficiency in DNA damage repair in BCBrM compared to ECT [11, 66].

Finally, and consistent with prior work, ECT exhibited higher expression of immune cell pathways compared to BrM, illustrating that the CNS environment around BrM is immunosuppressive [21, 27, 65]. Interestingly, when deconvoluting immune cell fractions, the only significant difference between ECT and BrM in our cohort was the dendritic cell population, with observed higher fractions in the BrM. A prior larger study by Giannoudis et al. of 26 patients with paired BCBrM-primary BC samples reported decreased dendritic cells in BCBrM compared to primary BC, but also reported significant differences in other populations, such as reductions in natural killer cells and mast cells and increases in B cells and neutrophils, which we did not observe in our smaller cohort [27]. Of note, there is a dendritic cell therapy in development for the treatment of BC leptomeningeal disease, with results anticipated in the next year (NCT05809752). Prior studies have demonstrated a reduction in tumor-infiltrating lymphocytes (TILs) and a general shift towards a more "immune cold" phenotype in BCBrM relative to primary BC [27]. While we did not detect differences in immune cell populations by intrinsic or clinical subtypes, likely due to our small sample size, prior studies have reported higher levels of microglia/macrophages in HR + HER2− and higher CD8 + granzyme B + T cell and TILs in TNBC BrM relative to other clinical subtype BCBrM [27, 28].

While not the primary focus of our study, it should be highlighted that there were some observed differences in PAM50 inferred subtypes between matched FFPE and frozen BrM. Inferred intrinsic subtypes were discordant in 23% of cases between FFPE and frozen BrM, with all discordant cases involving the LumB subtype, predominantly from FFPE samples. To our knowledge this is the first study to compare intrinsic molecular subtypes in matched FFPE and frozen BCBrM, though other studies have conducted similar comparisons in other BC specimens with similar results. A prior study assessed the TNBC molecular subtype concordance in 11 patients with RNA sequencing data on an Illumina HiSeq from matched FFPE and frozen TNBC primary tumor specimens [35]. That study reported that 9 of the 11 patient pairs yielded TNBC subtype calls in both the FFPE and frozen paired specimens, with 33% (3/9) of those patients exhibiting discordance between the two [35], similar to our observed rate of 23% for PAM50 calls. A second study compared intrinsic subtypes based on Single Sample Predicator (SSP) calls in microarray results from matched FFPE and fresh frozen primary BC tumors of 20 patients and found only a 5% (1/20) discordance rate between FFPE and frozen samples [56]. A third study specifically assessed PAM50-determined intrinsic subtypes in matched FFPE and frozen primary early breast cancer specimens from 94 patients, and reported an overall discordance rate of 20% (19/94), including 27% (15/54) of LumA FFPE, 6% (1/18) of LumB FFPE, and 33% (3/9) of HER2-E FFPE specimens being discordant with the matched frozen subtype call [46]. On a patient level, DNA alterations (somatic mutations and CNV) were generally similar between both preparation types, but there were individual cases where different mutations, or lack thereof, were observed in one sample, but not the other. RNA expression data did vary, sometimes significantly, between matched FFPE and frozen BrM, including in some CR genes, which could have contributed to differences observed in inferred subtypes and immune cell fractions in these pairs. Additional work to further define these differences is warranted to ensure biologic results, not technical results, inform treatments and prognosis.

There are several limitations to our study which inform interpretation of the results. First, our cohort was small and from a single institution, thus underpowered to detect significant differences in some of the analyses. Given the need to further stratify by intrinsic subtypes and sample types with matched pairs, a larger, multi-institutional study pooling data would be warranted. Further, one patient contributed two BrM samples and another contributed two ECT samples, though these were excluded in most analyses. Second, selection bias is applicable to our very select patient cohort: all patients had BrM; and specifically, likely had symptomatic and/or large BrM to warrant surgical removal. Thus, our results cannot be extrapolated to all patients with BC or BrM. Additionally, while our cohort had some clinical annotation, the information was collected retrospectively through review of Duke medical records, thus some information for patients was incomplete. For example, it is unknown if for the patients with matched FFPE and frozen BrM, if the samples came from the same region of one tumor, or from multiple regions or even different synchronous lesions. Treatment information prior to craniotomy was limited in our clinical records, thus we were unable to assess whether there was any association between prior exposure to systemic therapies and any genomic alterations in the BCBrM. Additionally, the immune cell fraction analysis is sensitive to the choice of signature matrix. Limited sample size also contributed to inflated *p* values in the time-to-event analyses (Additional file 1: Supplementary Fig. S12), and these inflated statistics were also used as the basis for the GSEA.

## Conclusions

Consistent with prior reports, in our cohort of 42 patients with BCBrM from all subtypes, we observed subtype concordance in the majority of matched FFPE BrM and FFPE ECT specimens, with discordances notably involving mostly the LumB subtype. As anticipated, both ECT and BrM tissues from BC exhibited some somatic alterations, with many more CNVs in genes, which varied by inferred subtype. Numerous genes and several pathways were differentially expressed between BrM and ECT, including MYC and E2F targets, oxidative phosphorylation, MTOR1 signaling, and DNA damage repair. The immune landscapes of ECT and BrM did not significantly differ between tissues or by subtype, aside from dendritic cells being overall higher in BrM. Each of these observations brings us one step closer to deciphering the genomic and immunobiology of breast cancer brain metastases to lead us toward superior therapies for this unique, yet growing, patient population.

## Supplementary Information

Supplementary Material 1. Supplementary Tables and Figures

Supplementary Material 2. Pathways of interest tested. 79 pathways of interest from Hallmark pathways, KEGG, Gene Ontology (GO), and literature were selected *a priori* and included pathways relevant to oxidative phosphorylation, MAPK interacting serine/threonine Kinase (MNK), cyclin dependent kinase (CDK), DNA damage repair, and immune signaling. Column names include: Pathway type, biologically-relevant pathway group name; Pathway name, unique pathway identifier; Number of genes, number of genes in pathway; Source, source of pathway.

Supplementary Material 3. Gene-wise summary of somatic and CNV alterations in clinically relevant and top 50 genes. Annotated details on the somatic and CNV alterations found in patients by gene. Column names include: Hugo_Symbol, HUGO gene symbol; nVariants, number of variants in gene; nSamples, number of samples with mutations in gene; nPatients, number of patients with mutations in gene.

Supplementary Material 4. Variant summary of somatic and CNV alterations in clinically relevant and top 50 genes. Annotated details on the somatic and CNV alterations found in patients by variant. For each tab in the workbook, column names include: BrM.PAM50, PAM50 inferred subtype (only shown in “All” tab which includes all samples across subtypes); Hugo_Symbol, HUGO gene symbol; Chromosome, chromosome in which variant is located; Start_Position, variant start position; End_Position, variant end position; Ref_Alt, reference and alternate alleles separated by “/”; Variant_Classification, VEP variant classification; nSamples, number of samples with variant; nPatients, number of patients with variant.

Supplementary Material 5. Differentially expressed genes in matched ECT-BrM specimens. Differentially expressed genes (DEGs) in matched FFPE ECT vs FFPE BrM (*n* = 8 patients), where log2 fold changes greater than 1 represent higher expression in BrM. DEGs are defined by having an adjusted p-value less than 0.05 and the table includes the top 50 up-regulated and top 50 down-regulated DEGs, sorted by unadjusted p-value. Column names include: Gene symbol, HUGO gene symbol; Base mean, mean of the normalized counts divided by size factors over all samples; Log2 Fold Change, log2 fold change in gene expression; Log2 Fold Change SE, standard error of the log2 fold change; Wald test statistic; P, unadjusted Wald test p value; Adjusted P, FDR adjusted p value; Direction, indicates if the gene is up- or down-regulated with respect to the BrM group.

Supplementary Material 6. Gene set enrichment analysis results based on HALLMARK pathways in matched ECT-BrM specimens. Results of gene set enrichment analysis (GSEA) based on HALLMARK pathways in matched FFPE ECT vs FFPE BrM (*n* = 8 patients), ranked by unadjusted *p* value. Column names include: Pathway, unique pathway identifier; P, unadjusted enrichment p value; Adjusted P, FDR adjusted *p* value; ES, enrichment score; NES, enrichment score normalized to mean enrichment of random samples of the same size; Number of genes, number of genes analyzed in the pathway; leadingEdge, HUGO gene symbols of leading-edge genes.

Supplementary Material 7. Gene set enrichment analysis results based on pathways of interest in matched ECT-BrM specimens. Results of gene set enrichment analysis (GSEA) based on selected pathways of interest (MNK, CDK, DNA damage repair, immune signaling, oxidative phosphorylation) from HALLMARK, KEGG, and GO gene lists and literature were compared in matched FFPE ECT-FFPE BrM specimens (*n* = 8 patients), ranked by unadjusted *p* value. Column names include: Pathway type, biologically-relevant pathway group name; Pathway, unique pathway identifier; P, unadjusted enrichment *p* value; Adjusted P, FDR adjusted *p* value; ES, enrichment score; NES, enrichment score normalized to mean enrichment of random samples of the same size; Number of genes, number of genes analyzed in the pathway; leadingEdge, HUGO gene symbols of leading-edge genes.

## Data Availability

All inferential analyses were carried out using the R Statistical Environment [63] along with extension packages from the Comprehensive R Archive Network (CRAN; https://cran.r-project.org/), including tidyverse v1.3.2 [80] and the Bioconductor project [34]. The analyses were carried out with adherence to the principles of reproducible analysis using the knitr v1.41 package [82] for generation of dynamic reports and using git for source code management. The code for replicating the statistical analysis was made available through a public source code repository (https://gitlab.oit.duke.edu/dcibioinformatics/pubs/anders-van-swearingen-bcbrm). The repository also provides the singularity [42] definition files for regenerating the computational environment used for this project. The patient-level source genomic and phenotypic data used in this project are available through dbGAP (phs003673.v1.p1).

## Abbreviations

Basal: Basal-like
BC: Breast cancer
BrM: Brain metastasis/metastases
CNS: Central nervous system
CNV: Copy number variation
ECT: Extracranial tumor
ER: Estrogen receptor
FFPE: Formalin-fixed paraffin embedded
GSEA: Gene set enrichment analysis
HER2: Human epidermal growth factor 2
HER2-Enr: HER2-Enriched
HR: Hormone receptor
LumA: Luminal A
LumB: Luminal B
MBC: Metastatic breast cancer
Normal: Normal-like
PR: Progesterone receptor
SNV: Single nucleotide variant
SRS: Stereotactic radiosurgery
TNBC: Triple negative breast cancer
WBRT: Whole brain radiotherapy
WES: Whole-exome sequencing
