# Supplementary material for: Genomic and immune profiling of breast cancer brain metastases
Source: Acta Neuropathol Commun. 2025 May 12;13:99. doi: 10.1186/s40478-025-02001-3 (PMC12070617; doi:10.1186/s40478-025-02001-3)
Supplement: Supplementary file 1 — Supplementary Material 1. Supplementary Tables and Figures [file 40478_2025_2001_MOESM1_ESM.docx]

**Supplementary Table S1: Sample cohort and post-craniotomy treatment overview.** Overview of the sample cohort, including breakdown by tissue (brain metastasis, BrM, vs extracranial tumor, ECT), processing (frozen vs formalin-fixed, paraffin-embedded, FFPE), and intrinsic subtype as determined by PAM50 analysis. Patient-matched blood DNA was collected to account for germline variation in tumor samples and improve somatic variant calling inference. A summary of post-craniotomy treatment history for the patient cohort is also included.

| **Sample Cohort** | | |
| --- | --- | --- |
|  | **Sample # (%)** | **Patient # (%)** |
| **DNA Samples** | 102 (100%) | 42 (100%) |
| Frozen BrM | 30 (29.4) | 29 (69.0%)^[[1]](#footnote-1)^ |
| FFPE BrM | 34 (33.3%) | 33 (78.6%)^1^ |
| FFPE ECT | 12 (11.8%) | 11 (26.2%)^[[2]](#footnote-2)^ |
| Patient-matched blood | 26 (25.5%) | 25 (59.5%)^[[3]](#footnote-3)^ |
|  |  |  |
| **RNA Samples and Intrinsic Subtypes (PAM50)** | 78 (100%) | 39 (100%) |
| Frozen BrM | 31 (39.7%) | 30 (76.9%)^1^ |
| Luminal A | 7 (22.6%) | 7 (23.3%) |
| Luminal B | 4 (12.9%) | 4 (13.3%) |
| HER2-enriched | 10 (32.3%) | 10 (33.3%) |
| Basal-like (including Claudin-low) | 9 (29.0%) | 8 (26.7%)^1^ |
| Normal-like | 1 (3.2%) | 1 (3.3%) |
| FFPE BrM | 34 (43.6%) | 33 (84.6%)^1^ |
| Luminal A | 5 (14.7%) | 5 (15.2%) |
| Luminal B | 8 (23.5%) | 8 (24.2%) |
| HER2-enriched | 11 (32.4%) | 11 (33.3%) |
| Basal-like (including Claudin-low) | 9 (26.5%) | 8 (24.2%)^1^ |
| Normal-like | 1 (2.9%) | 1 (3.0%) |
| FFPE ECT | 13 (16.7%) | 12 (30.8%)^2^ |
| Luminal A | 2 (15.4%) | 2 (16.7%) |
| Luminal B | 3 (23.1%) | 3 (25.0%) |
| HER2-enriched | 4 (30.8%) | 4 (33.3%)^2^ |
| Basal-like (including Claudin-low) | 3 (23.1%) | 3 (25.0%) |
| Normal-like | 1 (7.7%) | 1 (8.3%)^2^ |
|  |  | |
| **Treatment at Duke After Surgery of BrM** | 40 BrM^[[4]](#footnote-4)^ | |
| **Radiation Therapy** |  | |
| Yes | 31 (77.5%) | |
| WBRT | 7 (17.5%) | |
| SRS | 14 (35.0%) | |
| WBRT + SRS | 6 (15.0%) | |
| Other/Unspecified | 4 (10.0%) | |
| No | 7 (17.5%) | |
| Unknown | 2 (5.0%) | |
| **Systemic Therapy** |  | |
| Yes | 14 (35.0%) | |
| No | 24 (60.0) | |
| Unknown | 2 (5.0%) | |
| **Subsequent Surgery** |  | |
| Yes | 9 (22.5%) | |
| Subtotal Resection | 1 (2.5%) | |
| Gross Total Resection | 2 (5.0%) | |
| Biopsy | 2 (5.0%) | |
| Unspecified | 4 (10.0%) | |
| No | 29 (72.5%) | |
| Unknown | 2 (5.0%) | |

BrM: Brain Metastasis, ECT: Extracranial Tumor, ER: Estrogen Receptor, PR: Progesterone Receptor, HER2: human epidermal growth factor receptor 2, WBRT: Whole Brain Radiation Therapy, SRS: Stereotactic Radiosurgery, FFPE: Formalin-Fixed Paraffin-Embedded


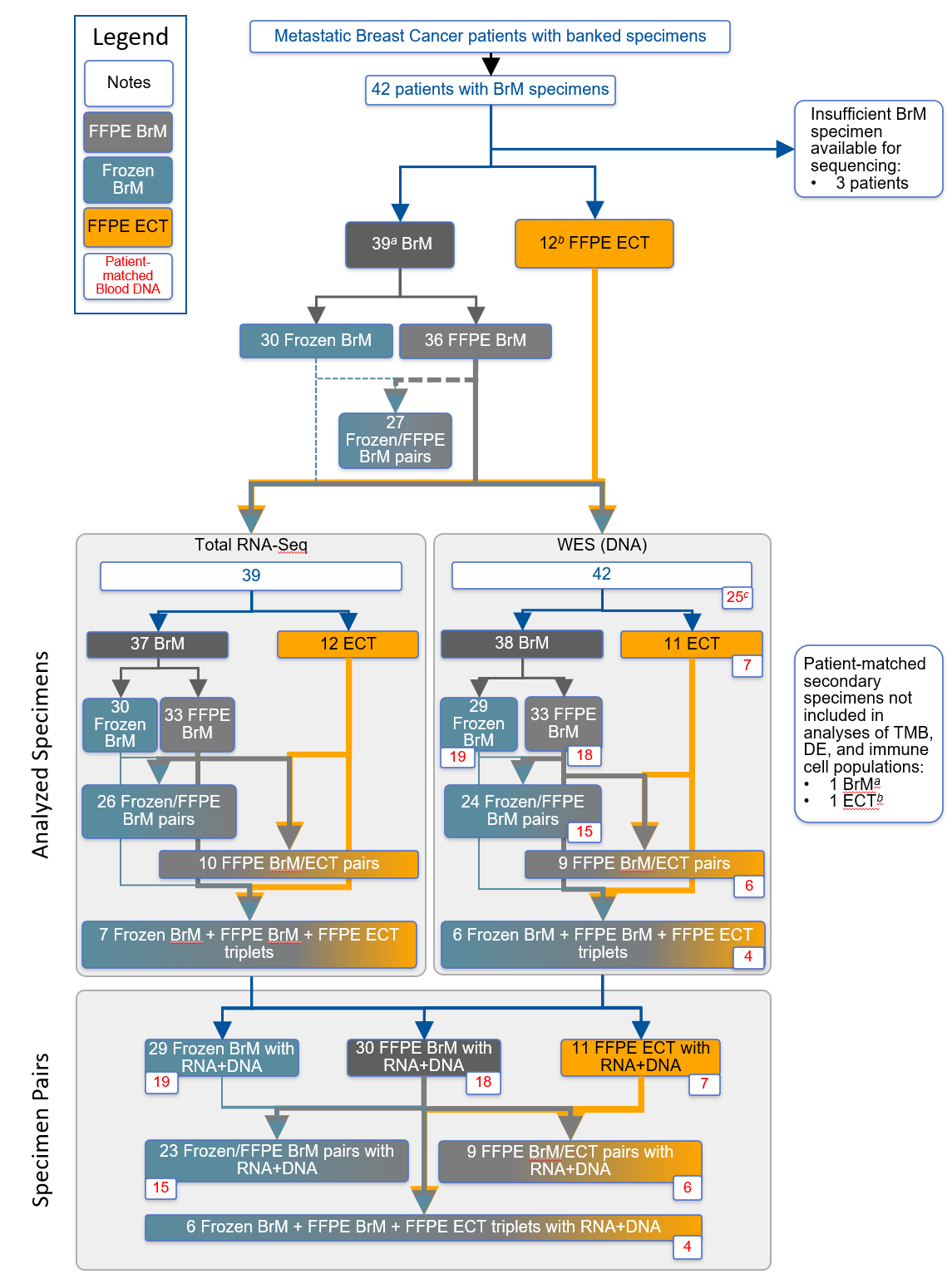


**Supplementary Figure S1: Consort diagram of patients represented in RNA and DNA samples analyzed.** Frozen and formalin-fixed, paraffin-embedded (FFPE) brain metastases (BrM), along with FFPE extracranial tumors (ECT) from a total of **42** patients with breast cancer BrM were included in the analyses. Patient-matched blood DNA was used to account for germline variation in tumor samples and improve somatic variant calling inference. Values in each text box reflect the number of patients in the group/pairing and red text boxes indicate patients with matched blood DNA available.

*^a^*: One patient had two asynchronous BrM resections roughly a year apart, both collected as FFPE and Frozen.

*^b^*: A different patient had two FFPE ECT specimens: a breast tumor and an unspecified extracranial tumor. Secondary specimen types in *^a^* and *^b^* for a single patient were not included in **paired** analyses.

*^c^*: The same patient as in ^a^ with two asynchronous BrM had a blood sample collected at the time of each resection, each of which was used to call somatic variants in each BrM sample.

**a**


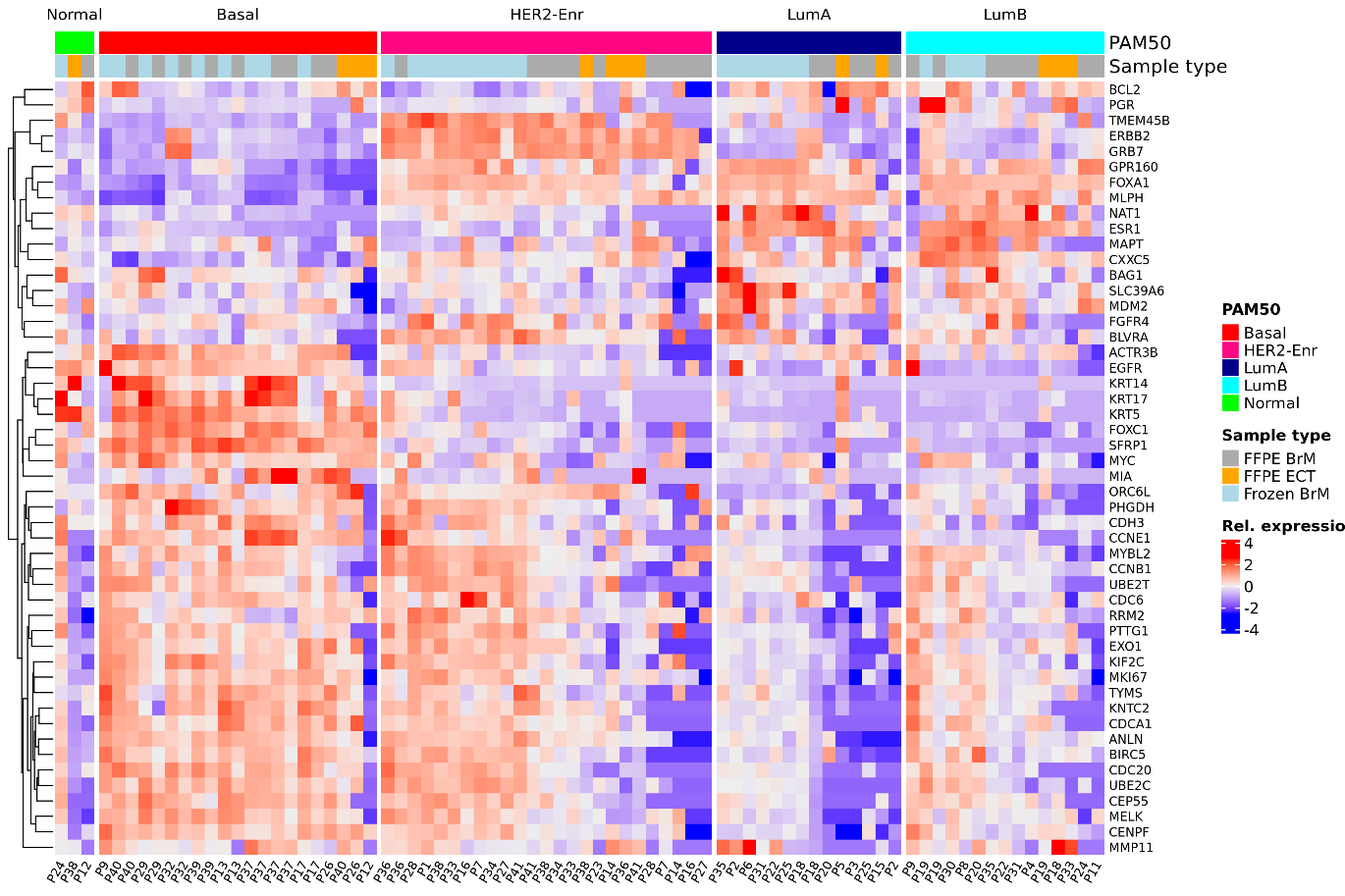


**b**


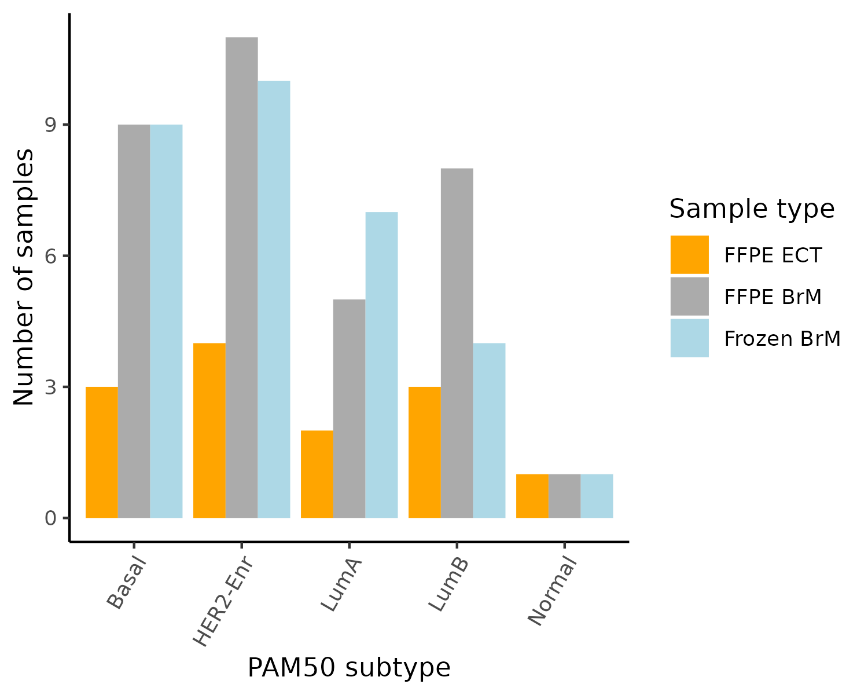


**Supplementary Figure S2: Intrinsic subtype gene expression and distribution of extracranial tumors and brain metastases. a)** Gene expression of intrinsic subtyping PAM50 genes within RNA samples (m=78 samples, n=39 patients). **b)** Distribution of PAM50 inferred intrinsic subtypes in RNA samples (m=78, n=39).


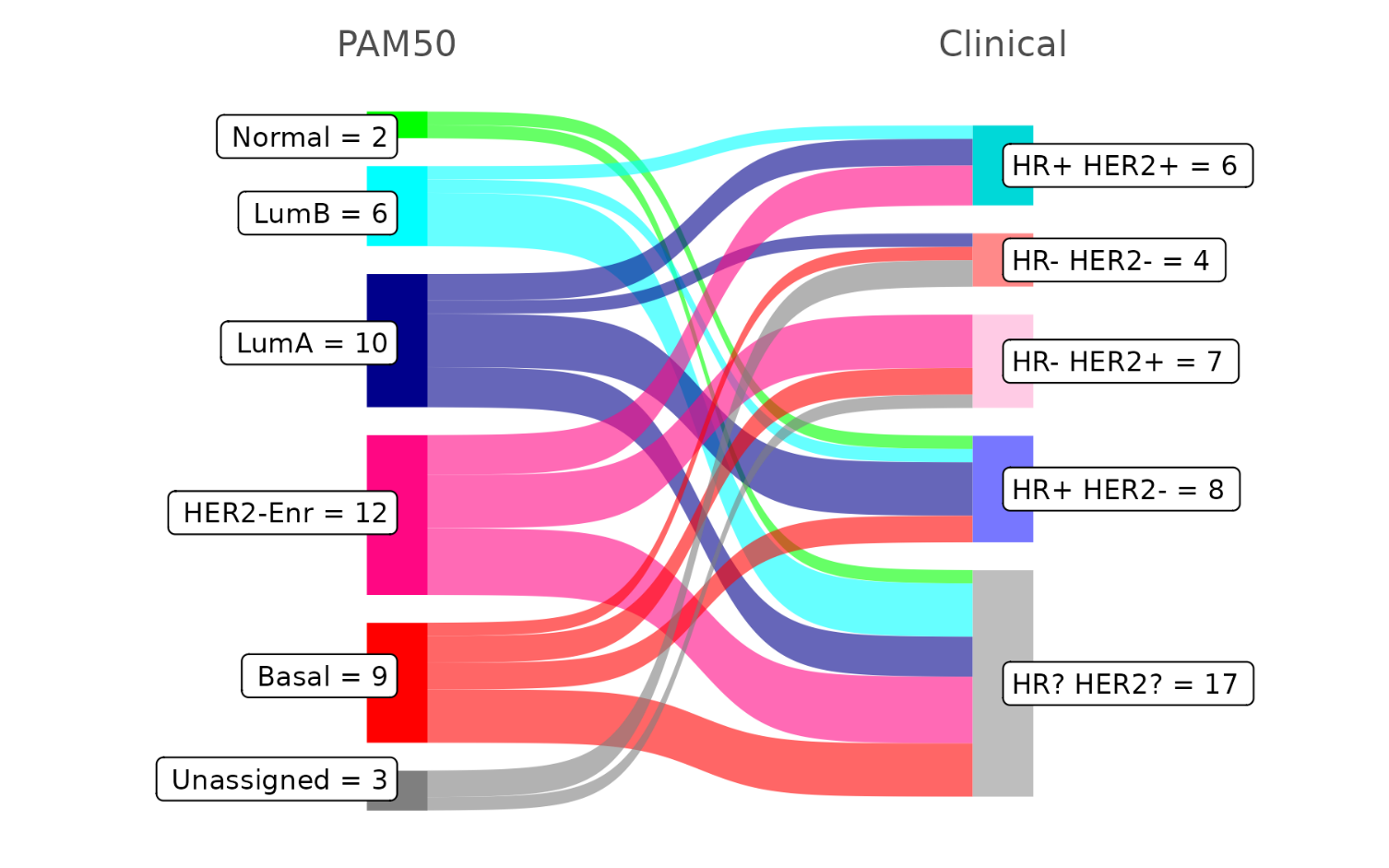


**Supplementary Figure S3: Intrinsic (PAM50) and clinical subtype concordance.** Concordance between a patient’s PAM50 inferred intrinsic subtype and their clinical subtype based on clinically assessed ER, PR, and HER2 immunohistochemistry. Values above represent number of patients (total n=42). To represent a patient’s PAM50 inferred subtype, calls from frozen BrM RNA samples were prioritized if available (n=30), followed by calls from FFPE BrM (n=7) or FFPE ECT samples (n=2), in that order; three patients have unassigned PAM50 subtype because they did not contribute RNA samples (n=3). Clinical subtype as determined by a BrM was only available for 25 patients, with the remaining classified as unknown (HR? HER2?) clinically.


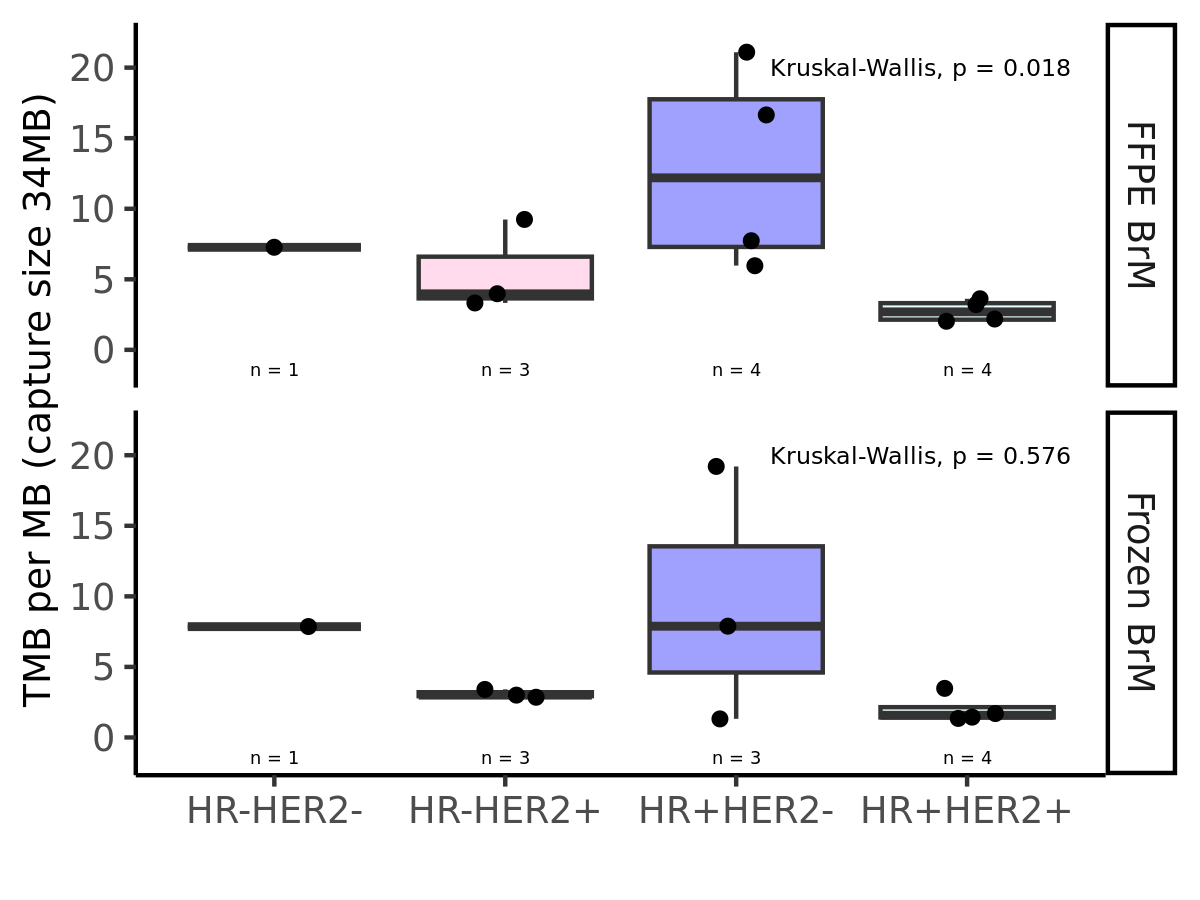


**Supplementary Figure S4: TMB across clinical subtypes.** TMB by clinical subtype for each sample type (FFPE BrM, n=12; Frozen BrM, n=11). Samples in the analysis were limited to those with clinical subtype designations determined in BrM. Secondary BrM and ECT samples are not shown nor analyzed. P-values shown in the figure are unadjusted.


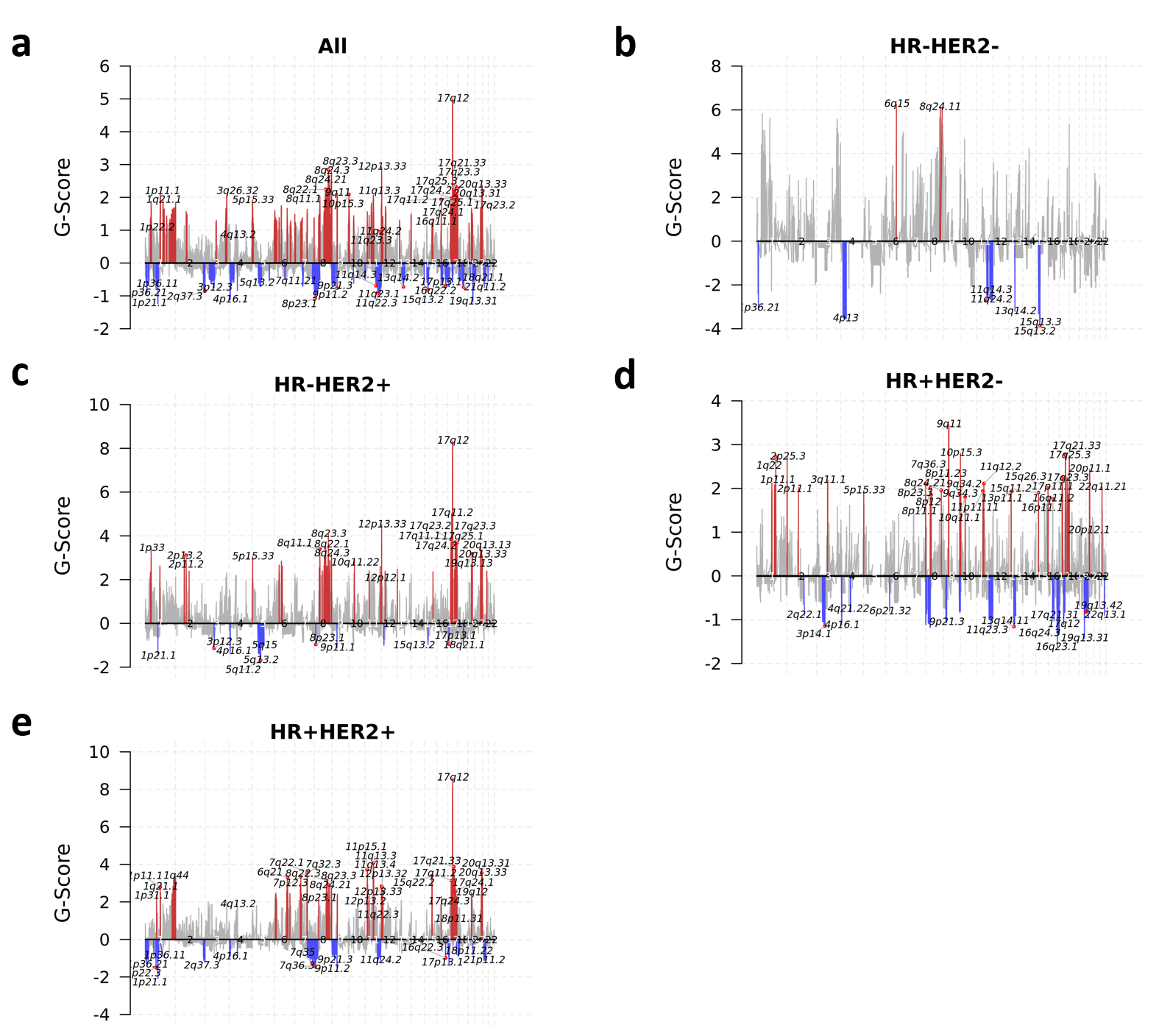


**Supplementary Figure S5: Copy number variants across BrM samples by clinical subtype**. Chromosomal map by GISTIC of CNV frequency and size by G-Score in **a)** all (n=22 patients, m=34 samples), **b)** HR-HER2- (FFPE BrM n=2, m=2; frozen BrM n=1, m=1), **c)** HR-HER2+ (FFPE BrM n=6, m=6; frozen BrM n=4, m=4), **d)** HR+HER2- (FFPE BrM n=6, m=6; frozen BrM n=4, m=4), and **e)** HR+HER2+ (FFPE BrM n=6, m=6; frozen BrM n=5, m=5). Gains/amplifications (red) are above the x axis, losses/deletions (blue) below the x axis, with significant (FDR<0.10) variants colored and the chromosome arm region annotated; grey peaks indicate gains/amplifications with FDR<0.25. Samples in the analysis were limited to those with clinical subtype designations determined in BrM.


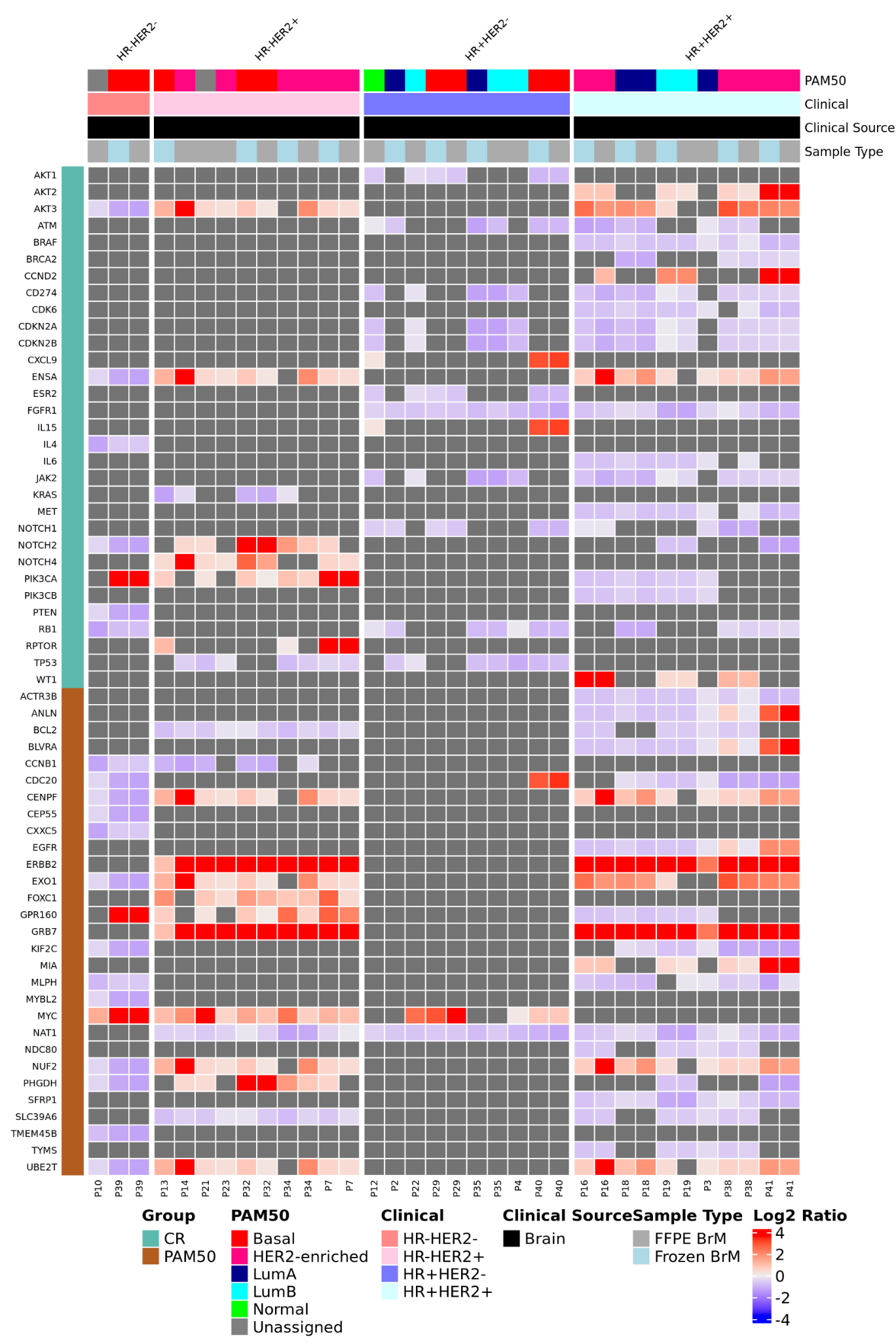


**Supplementary Figure S5 (continued): Copy number variants across BrM samples by clinical subtype. f)** Heatmap of log2 copy number ratios in CR and PAM50 genes overlapping significantly amplified/deleted regions identified by GISTIC2 (FDR<0.25) in FFPE BrM (n=20 patients, m=20 samples) and frozen BrM (n=14, m=14) by clinical subtype. For cases where there was more than one GISTIC peak region associated with a given gene symbol and sample, the longest peak region was selected to be included in the heatmap. Samples in the analysis were limited to those with clinical subtype designations determined in BrM.


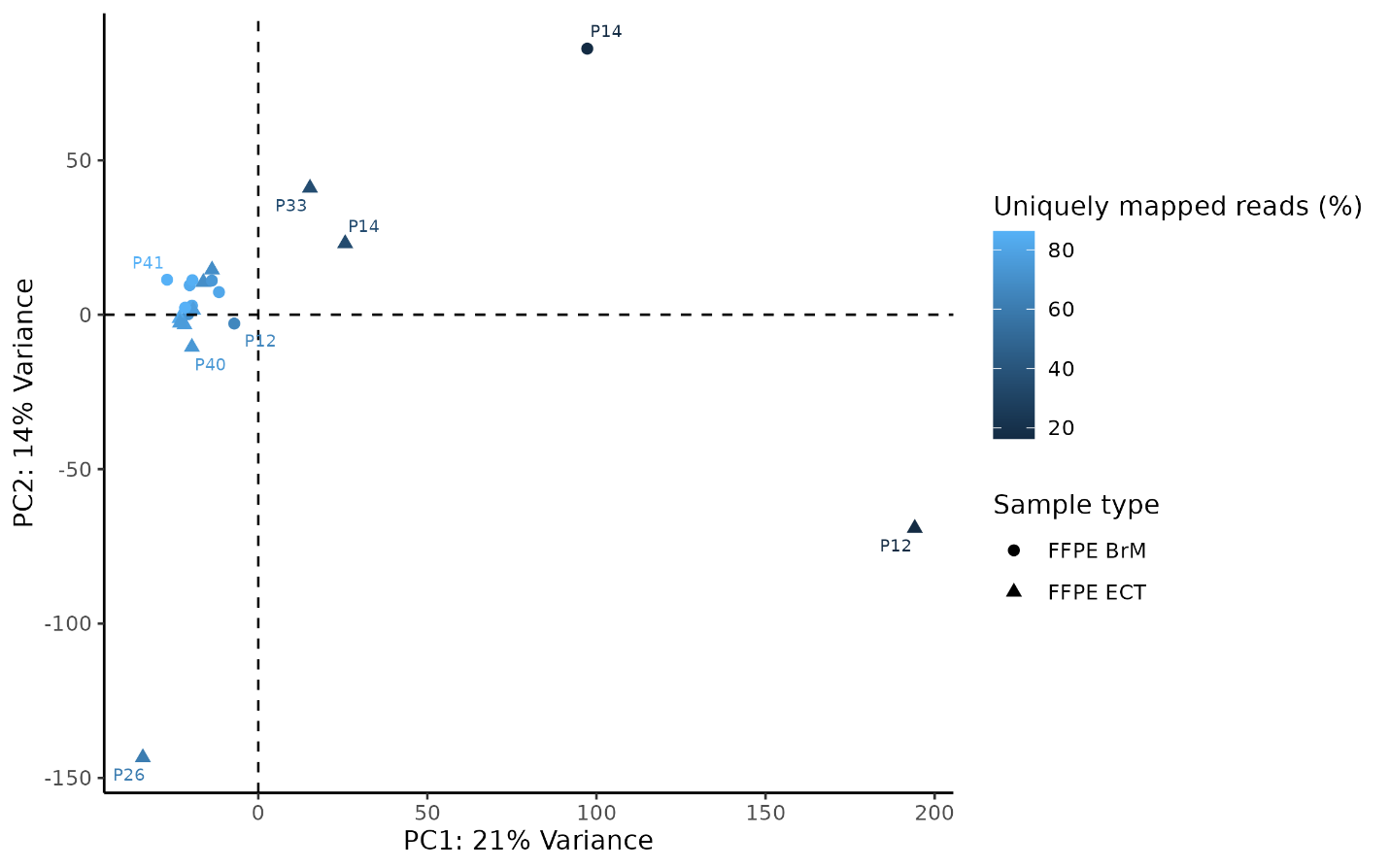


**Supplementary Figure S6: Principal component analysis of gene expression data in BrM and ECT.**PCA of gene expression from patient-paired FFPE BrM-FFPE ECT samples (n=10 patients, m=21 samples) with sample type indicated by point shape and the percentage of uniquely mapped reads indicated by blue shade. Two samples, both FFPE ECT, were excluded as outliers in gene expression and due to low sequencing quality based on the percentage of uniquely mapped reads. In addition, the secondary FFPE EC sample contributed by one patient was excluded.

**a**


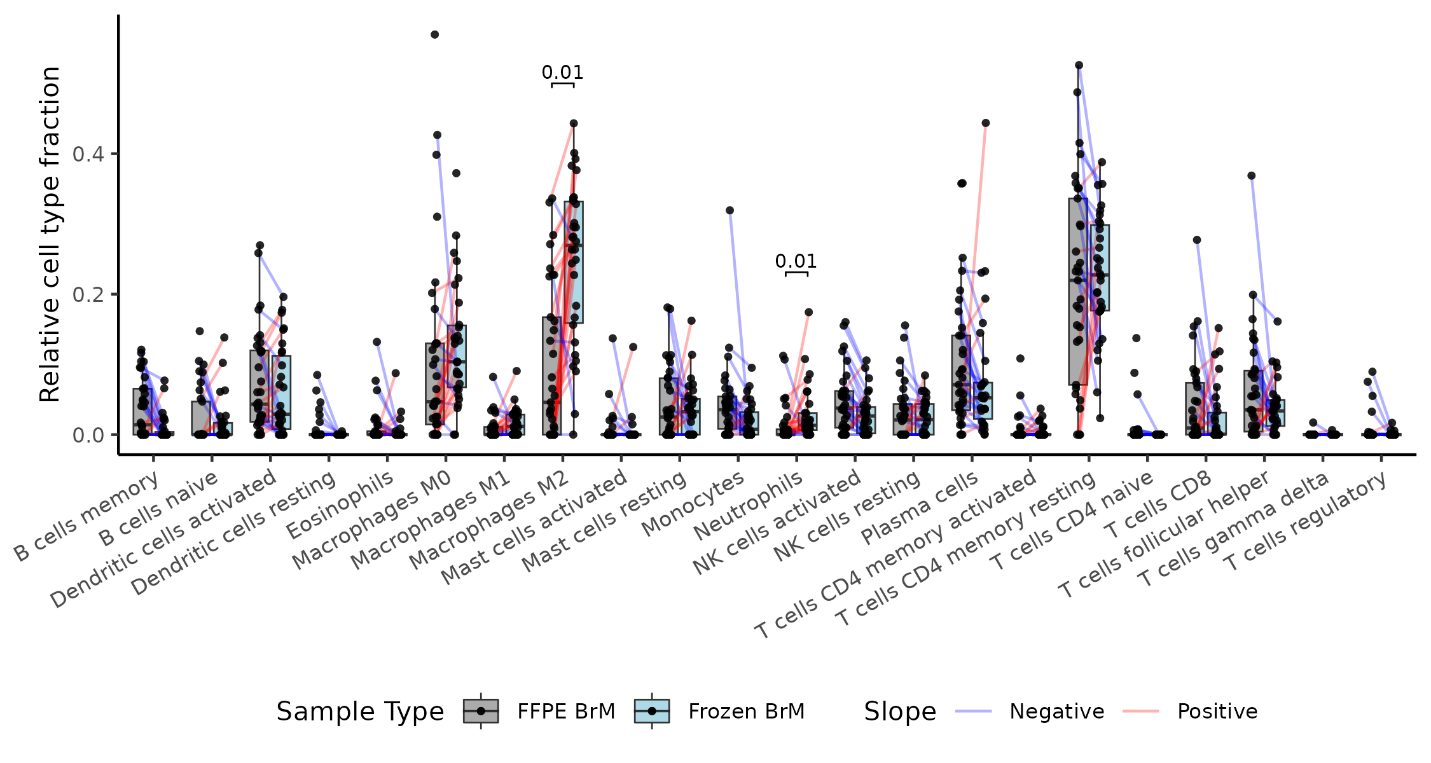


**b**


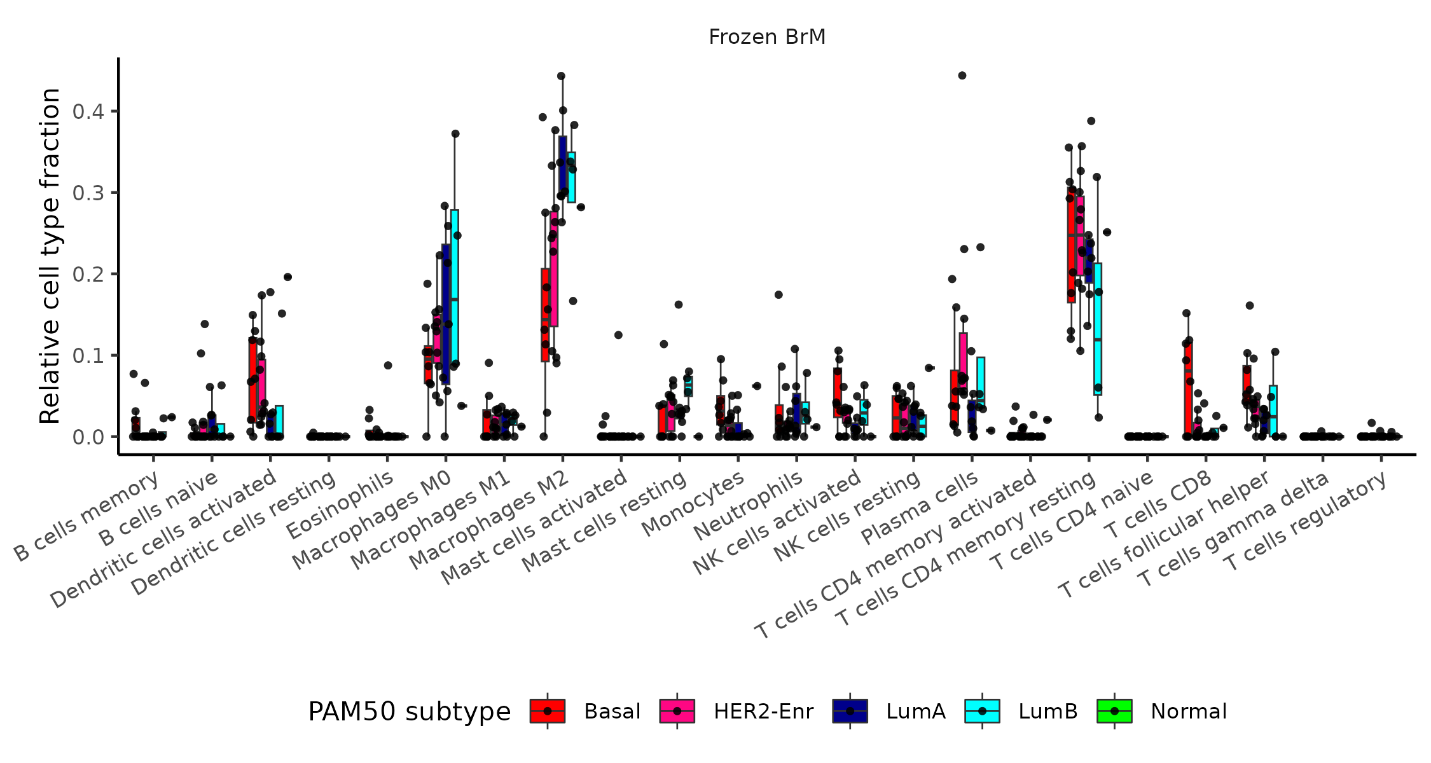


**Supplementary Figure S7: Deconvolved immune cell populations in brain metastases. a)** Fractions of immune cell populations inferred by CIBERSORTx in FFPE BrM (n=33 patients, m=33 samples) and frozen BrM (n=30, m=30). Patient-matched FFPE BrM and frozen BrM samples (n=26) are connected by lines colored by slope direction. Significant differences (adjusted p<0.05) are displayed between patient-matched FFPE BrM and frozen BrM cell fractions based on Wilcoxon signed-rank tests with p-values adjusted for testing multiple cell types. **b)** Fractions of immune cell populations inferred by CIBERSORTx by PAM50 subtype within each tissue type. No significant differences were detected between PAM50 inferred subtypes within a given sample type using Kruskal-Wallis tests with p-values adjusted for testing multiple cell types. Secondary BrM samples are not shown nor analyzed. PAM50 Normal samples (n=1, m=1) are shown in panel b but not analyzed.

**a**


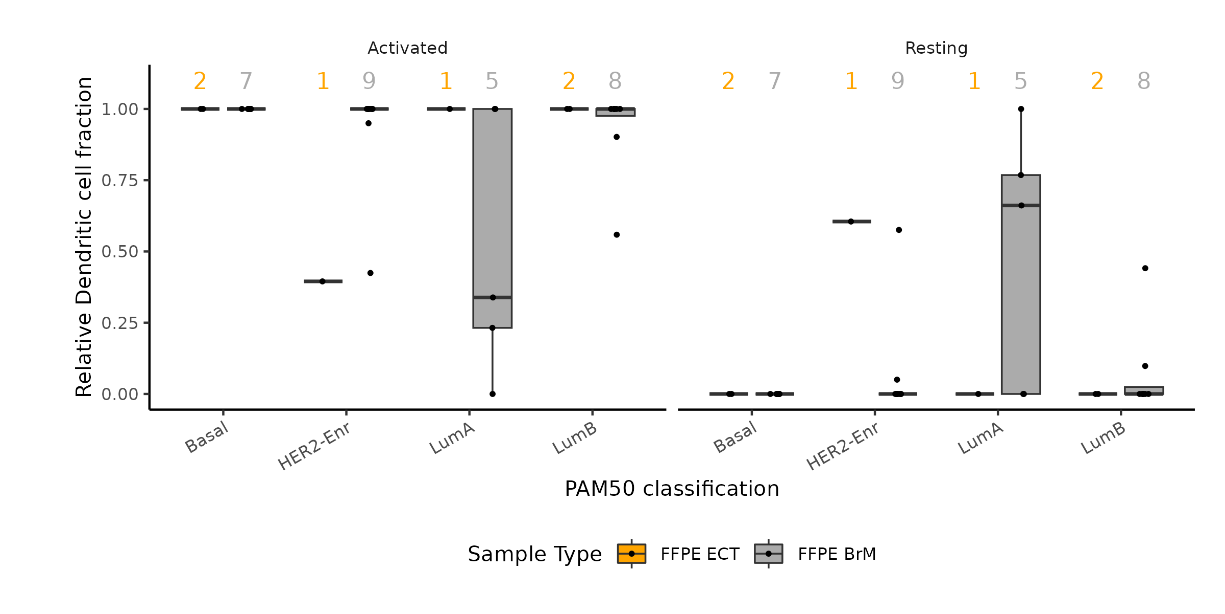


**b**


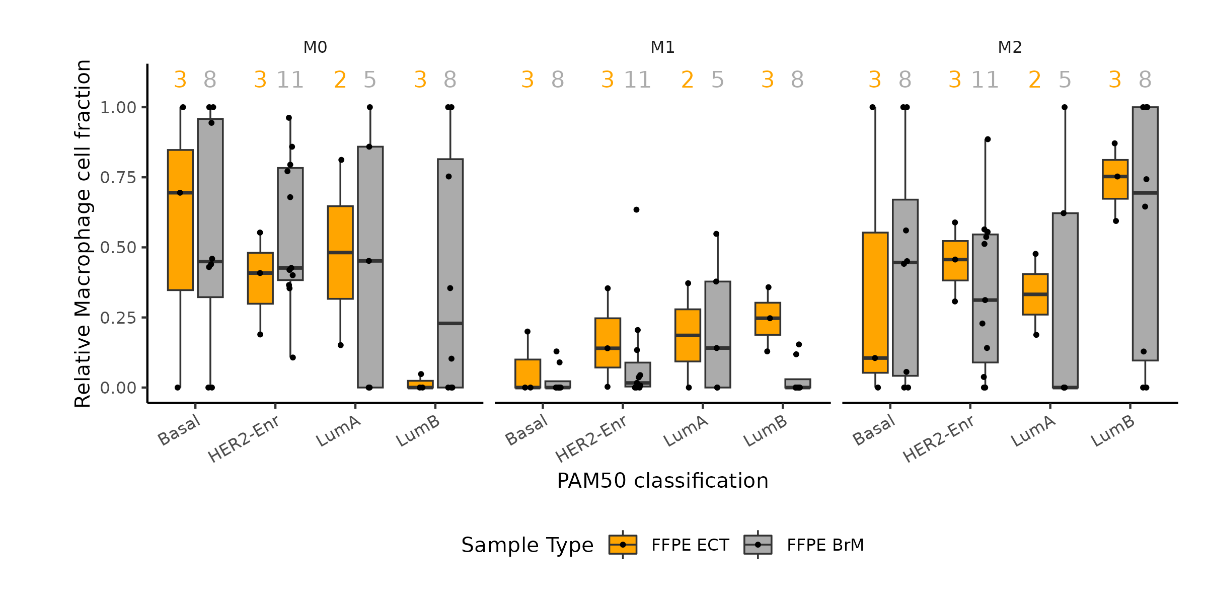


**c**


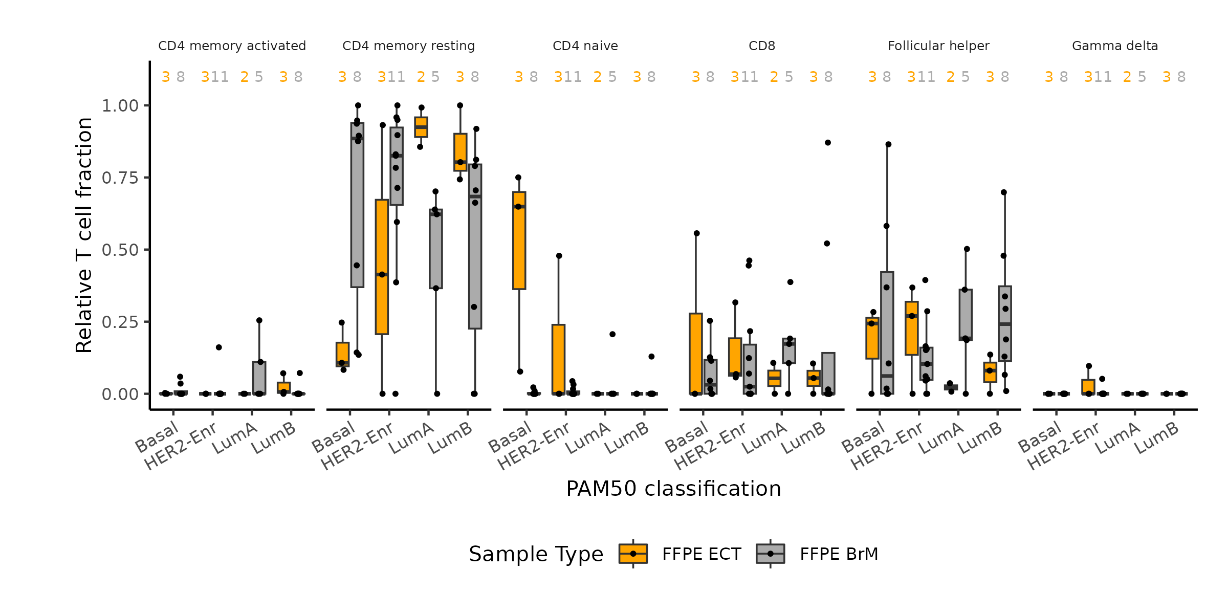


**Supplementary Figure S8: Relative fraction of immune cell populations within selected cell types in extracranial tumors and brain metastases.** Inferred sub-fractions of immune cell populations within **a)** dendritic cells, **b)** macrophages, and **c)** T cells by sample type and PAM50 inferred intrinsic subtype. Relative cell fraction is calculated as the target cell population fraction divided by the sum of all cell fractions in the cell type (i.e. M0/ (M0+M1+M2)). The 1 FFPE ECT and 1 FFPE BrM samples classified as PAM50 Normal are not displayed nor analyzed. Samples without any inferred dendritic cell fractions (FFPE ECT m=5, FFPE BrM m=3) are not shown. Text numbers above the bars indicate the number of samples per group, colored by sample type. Secondary BrM and ECT samples are not shown nor analyzed.

**a b**


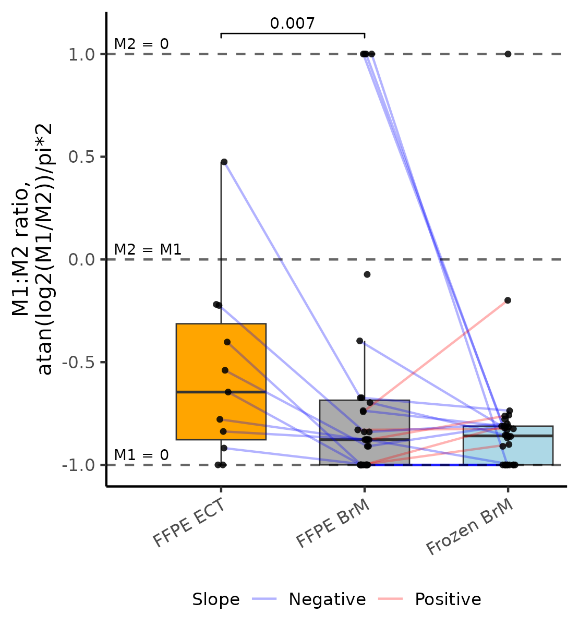

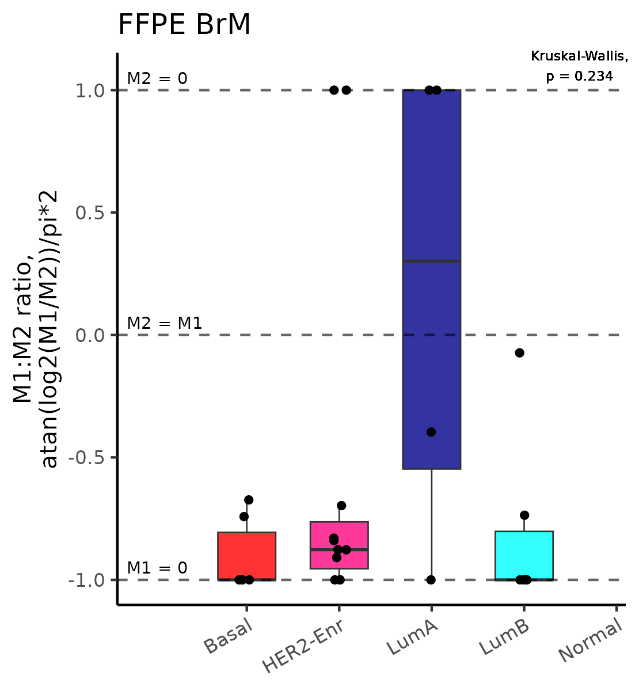


**c d**


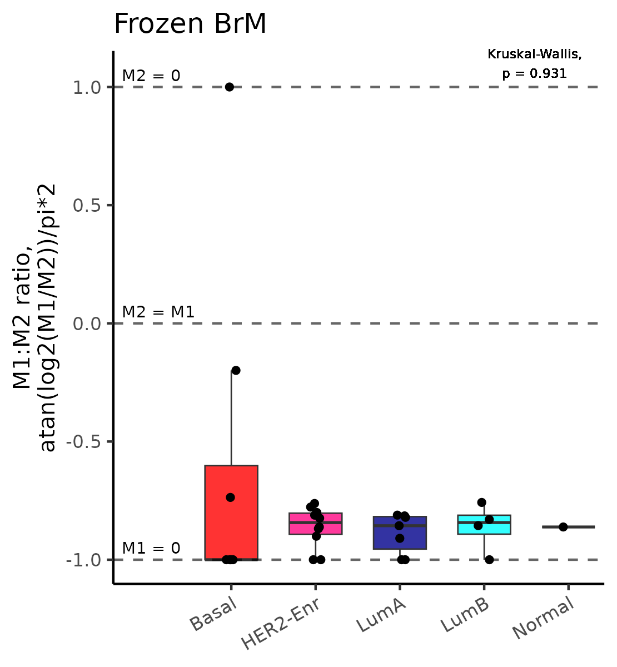

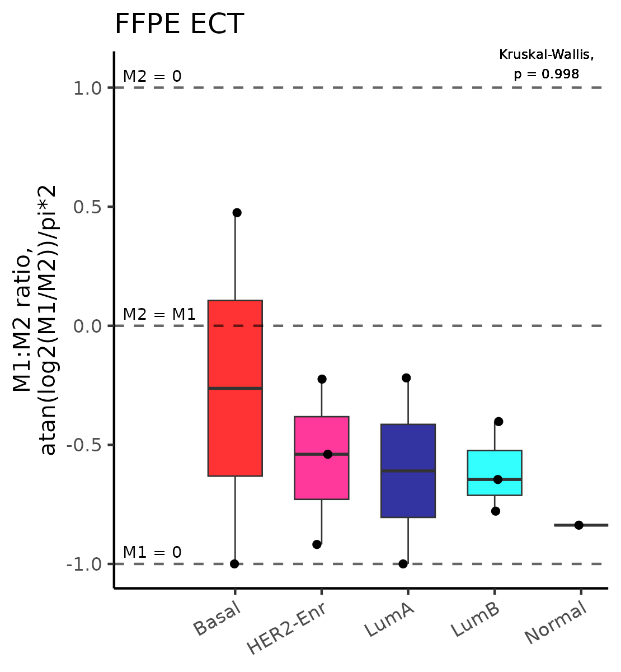


**Supplementary Figure S9: M1:M2 ratios of deconvoluted macrophage populations in extracranial tumors and brain metastases.** Ratio of M1:M2 macrophage fractions inferred by CIBERSORTx in a) FFPE ECT (n=11 patients, m=11 samples), FFPE BrM (n=27, m=27), and frozen BrM (n=30, m=30) samples, and within each sample type by PAM50 inferred intrinsic subtype for b) FFPE BrM, c) frozen BrM, and d) FFPE ECT. Samples without any inferred M1 or M2 fractions (FFPE ECT m=1, FFPE BrM m=6) are not shown. Patient-matched FFPE BrM and FFPE ECT samples (n=8) and FFPE BrM and frozen BrM samples (n=21) are connected by lines colored by slope direction. Significant differences (adjusted p<0.05) are displayed between patient-matched fractions based on Wilcoxon signed-rank tests. No significant differences were detected between PAM50 inferred subtypes within a given sample type using Kruskal-Wallis tests. Secondary BrM and ECT samples are not shown nor analyzed.

**a**
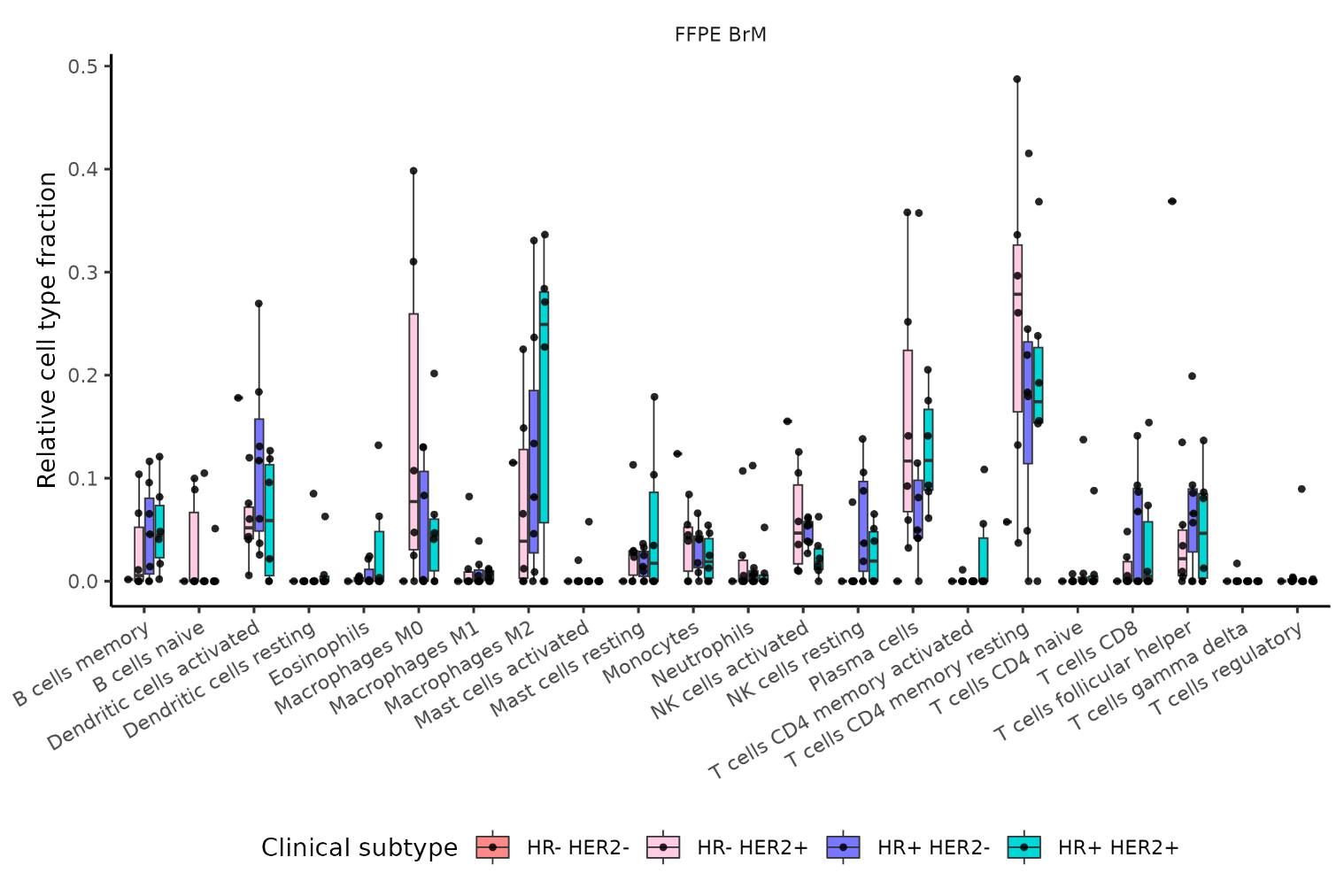
 **b**
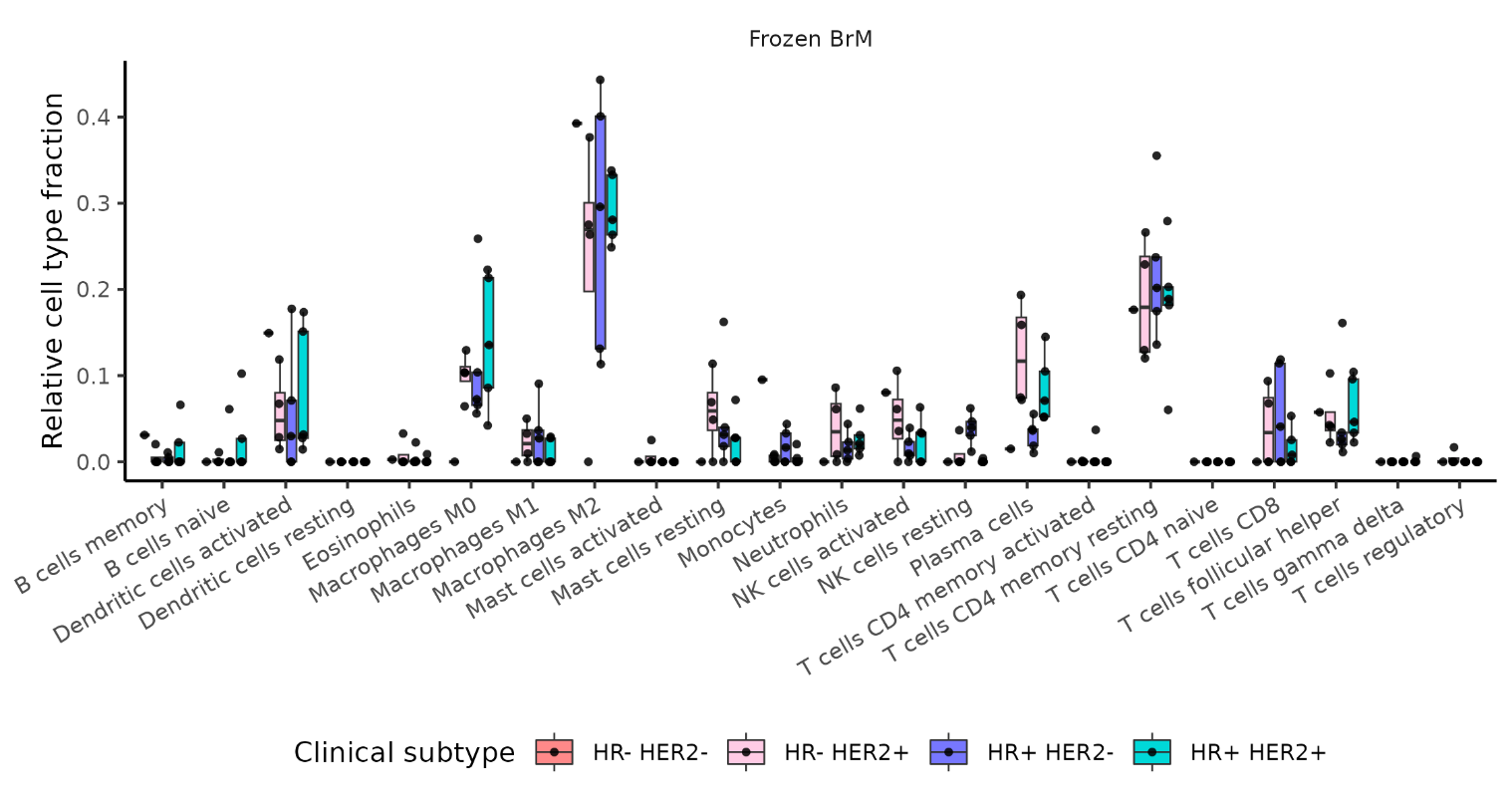


**Supplementary Figure S10: Deconvolved immune cell populations in FFPE brain metastases by clinical subtype.** Fractions of immune cell populations inferred by CIBERSORTx by clinical subtype within **a)** FFPE BrM (HR-HER2- n=1, HR-HER2+ n=6, HR+HER2- n=7, HR+HER2+ n=6) and **b)** Frozen BrM (HR-HER2- n=1, HR-HER2+ n=4, HR+HER2- n=5, HR+HER2+ n=5) sample types. No significant differences were detected between clinical subtypes within a given sample type using Kruskal-Wallis tests with p-values adjusted for testing multiple cell types. Clinical subtype designations are limited to those determined by BrM. Secondary BrM samples are not shown nor analyzed.

**a DNA Repair b Inflammatory Response**

**
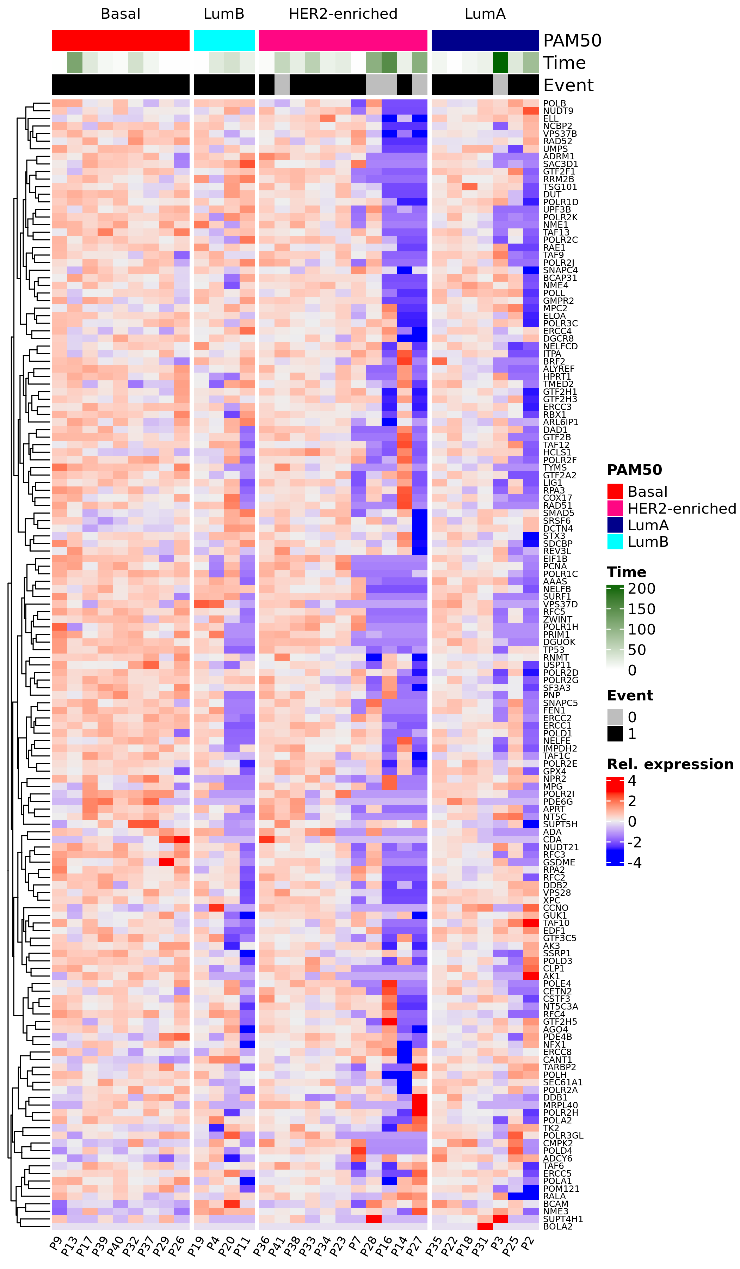

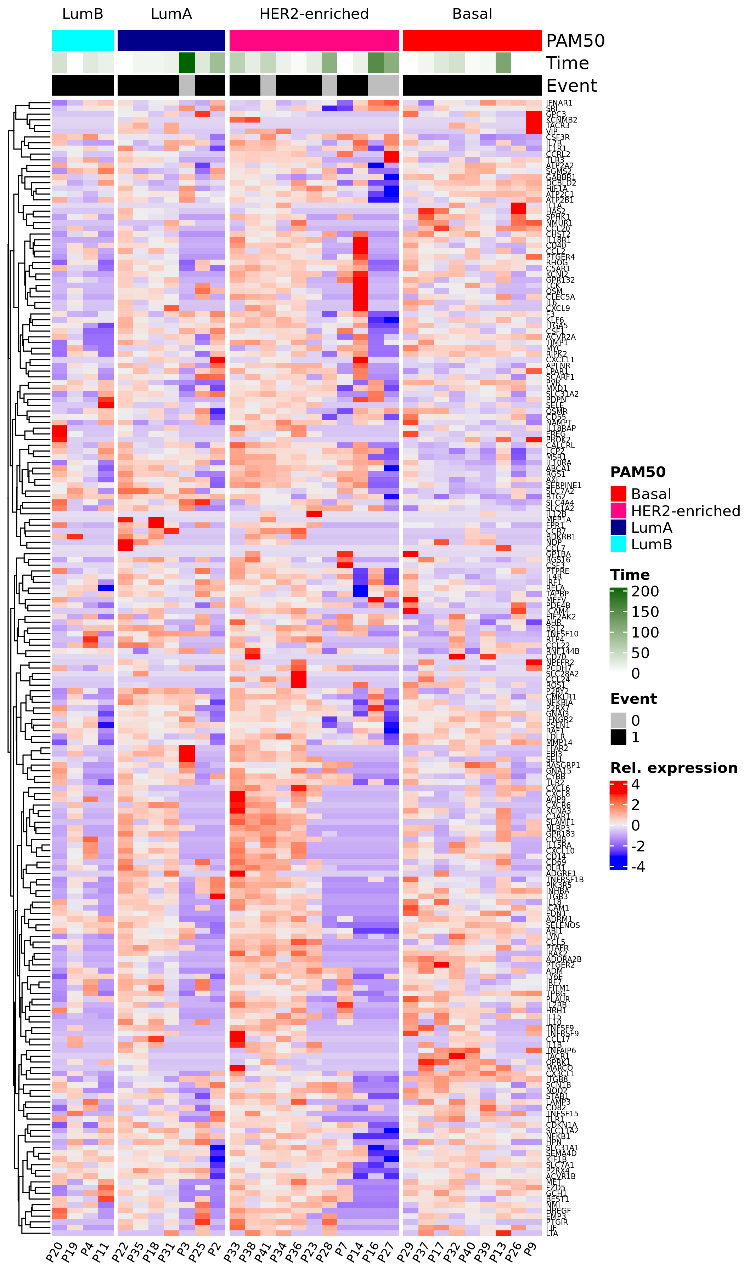
**

**Supplementary Figure S11: Heatmap of FFPE BrM pathways associated with time from craniotomy to death or last follow-up.** The analysis included 39 patients with FFPE BrMs. Two PAM50 Normal BrM samples were excluded due to small sample size , and the secondary BrM sample from the patient that contributed two BrM is not shown nor analyzed.


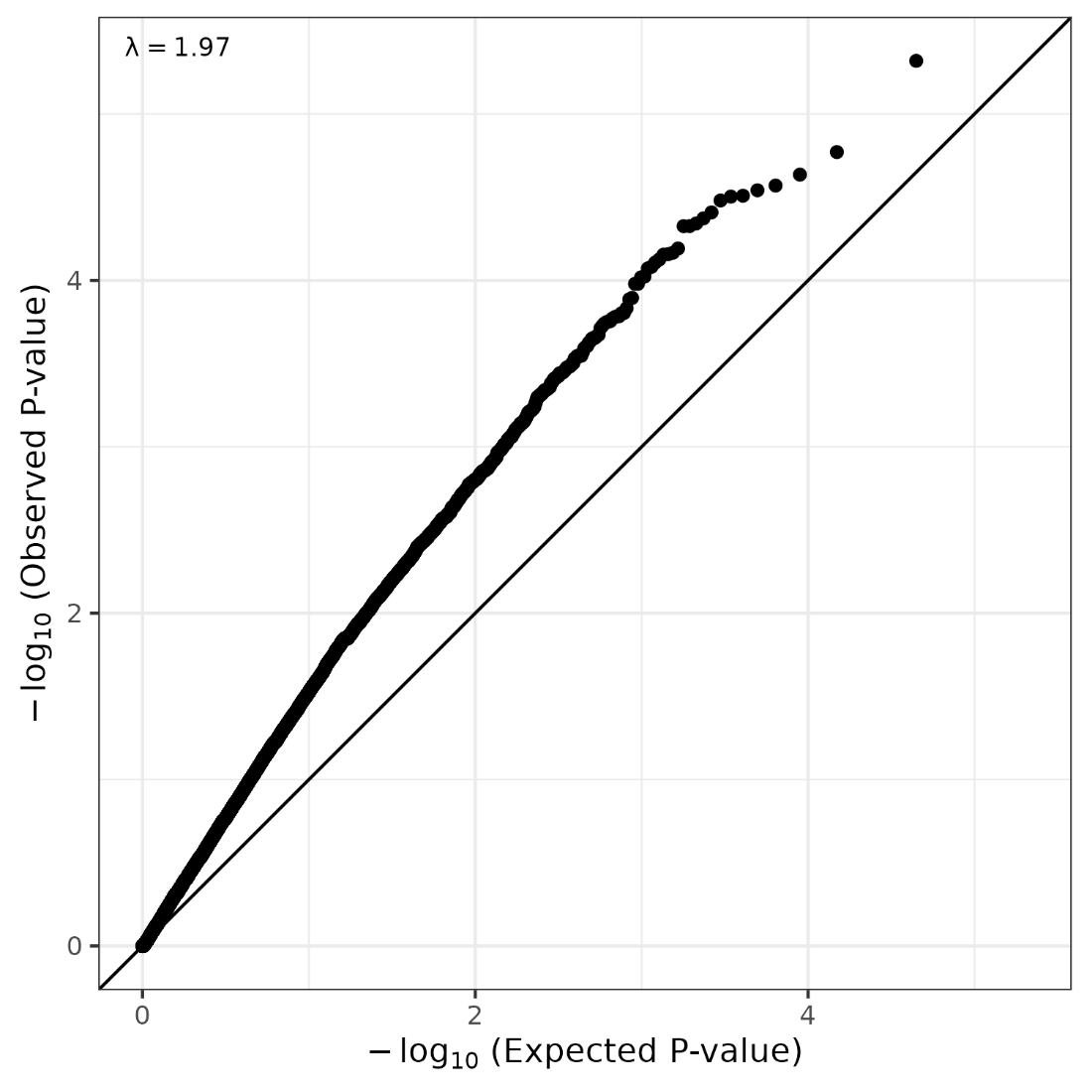


**Supplementary Figure S12: Geneset survival analysis quantile-quantile plot.** Expected versus observed P values from gene-level time-to-event Cox PH models (n=33) suggest that the P values are inflated.  These inflated statistics were also used as the basis for the time from craniotomy to death or last follow-up GSEA.

**a b**


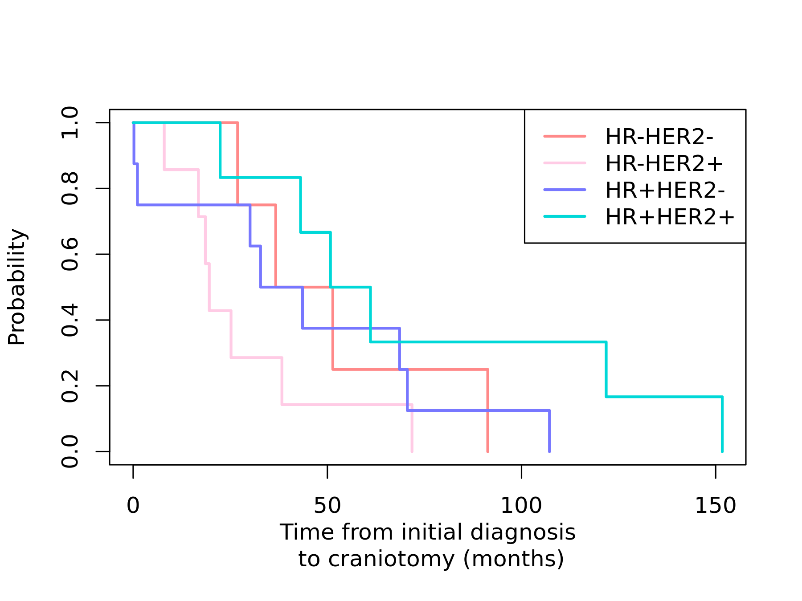

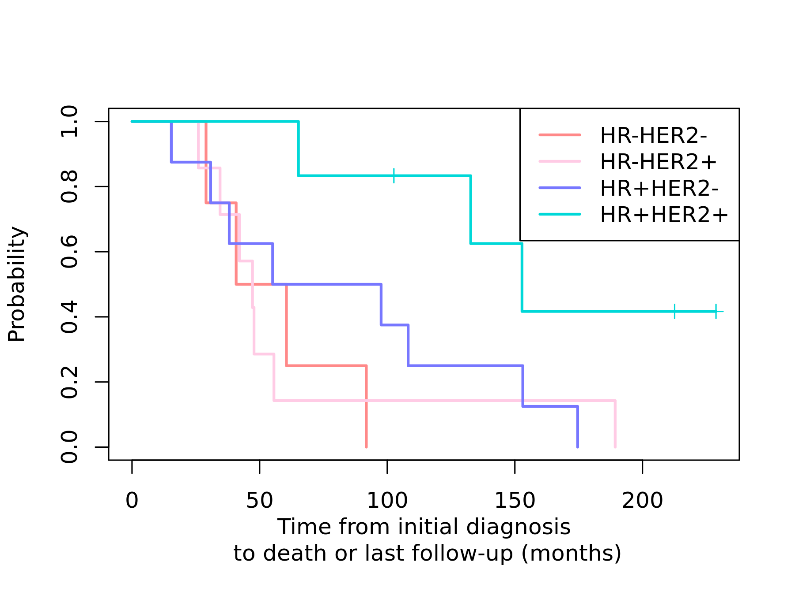
 **c**


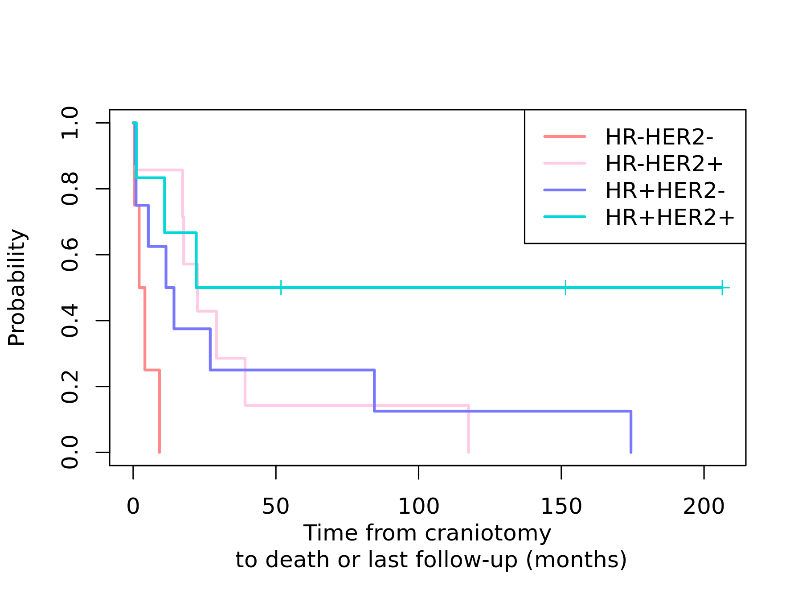


**Supplementary Figure S13: Clinical outcomes of patient cohort by clinical subtype.** Kaplan-Meyer plots of times to events (months) by clinical subtype for time from **a)** initial breast cancer diagnosis to craniotomy (not significant), **b)** initial BC diagnosis to death or last follow-up (unadjusted p=0.045, n=25) and **c)** craniotomy to death/last follow up (unadjusted p=0.009, n=25). Cases are limited to those with clinical subtype determined in BrM.

1. One patient had two asynchronous BrM resections roughly a year apart, both collected as FFPE and Frozen, all four samples were classified as Basal-like. [↑](#footnote-ref-1)
2. One patient had two FFPE ECTs: a PAM50-Normal-like breast tumor and a HER2-enriched unspecified tumor. [↑](#footnote-ref-2)
3. For the two asynchronous BrM resections from one patient, blood samples collected at the time of each resection were available and used to call somatic variants in each BrM sample. [↑](#footnote-ref-3)
4. Specimens were collected from 43 surgeries across 42 patients. Of the 43 surgeries, three of the BrM specimens were not available for sequencing, resulting in 40 BrM specimens. [↑](#footnote-ref-4)
